# Multi‐Color Luminescence Transition of Upconversion Nanocrystals via Crystal Phase Control with SiO_2_ for High Temperature Thermal Labels

**DOI:** 10.1002/advs.202000104

**Published:** 2020-04-24

**Authors:** Dahye Baek, Tae Kyung Lee, Inkyu Jeon, Se Hun Joo, Subeen Shin, Jaehyun Park, Seok Ju Kang, Sang Kyu Kwak, Jiseok Lee

**Affiliations:** ^1^ School of Energy and Chemical Engineering Ulsan National Institute of Science and Technology (UNIST) Ulsan 44919 Republic of Korea

**Keywords:** hexagonal apatite, luminescence transitions, optical labeling systems, phase transitions, upconversion nanocrystals

## Abstract

Upconversion nanocrystals (UCNs)‐embedded microarchitectures with luminescence color transition capability and enhanced luminescence intensity under extreme conditions are suitable for developing a robust labeling system in a high‐temperature thermal industrial process. However, most UCNs based labeling systems are limited by the loss of luminescence owing to the destruction of the crystalline phase or by a predetermined luminescence color without color transition capability. Herein, an unusual crystal phase transition of UCNs to a hexagonal apatite phase in the presence of SiO_2_ nanoparticles is reported with the enhancements of 130‐fold green luminescence and 52‐fold luminance as compared to that of the SiO_2_‐free counterpart. By rationally combining this strategy with an additive color mixing method using a mask‐less flow lithography technique, single to multiple luminescence color transition, scalable labeling systems with hidden letters‐, and multi‐luminescence colored microparticles are demonstrated for a UCNs luminescence color change‐based high temperature labeling system.

## Graphical abstract

The multiple luminescence color tuning of upconversion nanocrystals (UCNs) with enhanced intensity is realized via a SiO_2_‐NPs involved unique crystal phase transition method. As a proof of concept demonstration, the robust optical labeling system through stepwise alteration of the luminescence color or hidden pattern generation in UCNs embedded microarchitectures are suggested.

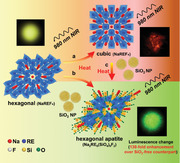

Spectrally tunable microarchitectures are effective information media because they act as carriers of luminescent materials that change their properties in response to external stimuli.^[^ ^1^, ^2^, ^3^, ^4^, ^5^, ^6^, ^7^, ^8^, ^9^, ^10^, ^11^, ^12^, ^13^, ^14^, ^15^, ^16^, ^17^, ^18^, ^19^, ^20^ ^]^ However, it is challenging to meet the stringent requirements of practical applications, such as in optical labeling systems under high‐temperature conditions. Thus, robust optical system with high thermal stability is required to reliably ensure product identification and the valuable information under high‐temperature thermal processes such as sterilization and cleansing of the steel, ceramic,^[^ ^21^ ^]^ and aerospace industries. The required temperature for labeling such products ranges from 600 up to 1600 °C, and the optical labeling system should survive these conditions for several hours. Downconversion ceramic materials are actively researched for practical applications due to their robust luminescence properties, but the ultra‐high temperature (1000–1550 °C) pre‐annealing process for the production of application systems is not suitable for actual industrial applications, and the system production cost is also very high because they require multiple optical filters to selectively observe the emission colors.^[^ ^22^, ^23^ ^]^ Recently, taking advantage of the technological advances in microfabrication techniques and the luminescence properties of upconversion nanocrystals (UCNs; e.g., anti‐Stokes effect,^[^ ^24^, ^25^, ^26^ ^]^ high photochemical stability,^[^ ^11^, ^27^, ^28^, ^29^, ^30^, ^31^ ^]^ and the absence of background autofluorescence^[^ ^27^, ^32^, ^33^, ^34^ ^]^), UCNs‐based labeling systems that can deliver the desired information in micro‐sized solid architectures have been developed in a scalable manner. Examples of such systems include latent fingerprinting,^[^ ^7^ ^]^ quick response codes,^[^ ^8^, ^9^, ^10^ ^]^ anti‐counterfeiting systems,^[^ ^3^, ^10^, ^11^, ^12^, ^20^ ^]^ and luminescence displays.^[^ ^13^, ^14^, ^15^ ^]^ The use of upconversion microparticles as a multiplexed labeling system overcomes many of the current limitations, such as a low information storage capacity,^[^ ^16^ ^]^ the requirement for costly decoders,^[^ ^17^, ^18^, ^19^ ^]^ and precise loading of optical dyes.^[^ ^2^, ^9^, ^17^, ^19^ ^]^ Many researches have been conducted to improve the inefficient upconversion process of UCNs by using surface plasmon resonance (SPR) of metal nanostructure^[^ ^35^, ^36^ ^]^ and by increasing the radiative decay rate of the emitters (Purcell effect),^[^ ^37^ ^]^ however, a majority of UCNs systems have a critical limitation in that they lose their luminescence property and change their multiple luminescence colors to weak red upon exposure to high temperature environments owing to the collapse of their inherent crystal structures.^[^ ^38^, ^39^ ^]^ Thus, a facile multiple luminescence color transition strategy that shows enhanced luminescence intensities and solid reading ability in a high temperature range would be desirable for the practical application.

Herein, we report a strategy to modulate the predetermined luminescence color of lanthanide‐ion doped UCNs with large enhancement in luminescence intensity upon heating with silica nanoparticles (SiO_2_ NPs). The key idea behind this work is incorporating SiO_2_ NPs into the crystal lattice of lanthanide‐ion doped UCNs at an elevated temperature to create a new crystal phase, hexagonal apatite, which has higher upconversion efficiency. The resulting hexagonal apatite UCNs show enhanced emission intensity, ideally higher than that of the SiO_2_ NPs‐free counterpart. Extending the use of this optical property changing principle, we propose an optical labeling system with cryptographic multiple luminescence color, patterns, and letters for commercial high‐temperature thermal processes by combining an additive color mixing method and a digital micro‐mirror device (DMD)‐based mask‐less lithography technique.

**Figure** **1** illustrates the phase‐transition strategy for developing lanthanide‐doped UCNs with enhanced multi‐spectral luminescence intensity via a SiO_2_ NPs involved annealing process. In this study, we prepared hexagonal rod‐shaped yellow emissive UCNs by a conventional hydrothermal method^[^ ^13^ ^]^ (see Experimental Section for details and Note S1, Supporting Information). We studied three different phase transition processes: hexagonal NaREF_4_ to cubic NaREF_4_ phase with low upconversion efficiency (yellow to red), hexagonal NaREF_4_ to hexagonal apatite for achieving enhanced luminescence intensity (yellow to green), and cubic NaREF_4_ to hexagonal apatite to change the low‐efficiency red to high‐efficiency green luminescence. Note that RE indicates rare earth elements (e.g., Y, Gd, Yb, Er, and Tm). In the case of yellow emissive UCNs, the hexagonal‐to‐cubic phase transition results in a decrease of the upconversion luminescence at 550 nm (color from yellow to red) and lowering of the luminescence efficiency (Figure 1a), which is consistent with previous results.^[^ ^38^, ^39^ ^]^ In contrast, UCNs including SiO_2_ NPs converts to hexagonal apatite phase with large enhancement of the luminescence intensity at 550 nm compared to that of the annealed cubic UCNs (Figure 1b). Therefore, in the case of cubic to hexagonal apatite phase transition, we reason that SiO_2_ NPs might enable the recuperation of the cubic phase UCNs to the hexagonal apatite phase accompanied by enhanced luminescence efficiency (Figure 1c). We studied various types of metalloid oxides (Al_2_O_3,_ Sb_2_O_3_, As_2_O_3_, GeO_2_, and SiO_2_) and they exhibited enhanced luminescence intensity upon the same thermal process (Figure S2, Supporting Information). SiO_2_ NPs are the most cost‐effective, easy‐to‐use material and showed most uniform dispersion property in the photocurable resin used in this study. Therefore, for a precise quantitative optical analysis of the investigated unique crystal phase transition, all further experiments were conducted using SiO_2_ NPs.

**Figure 1:**
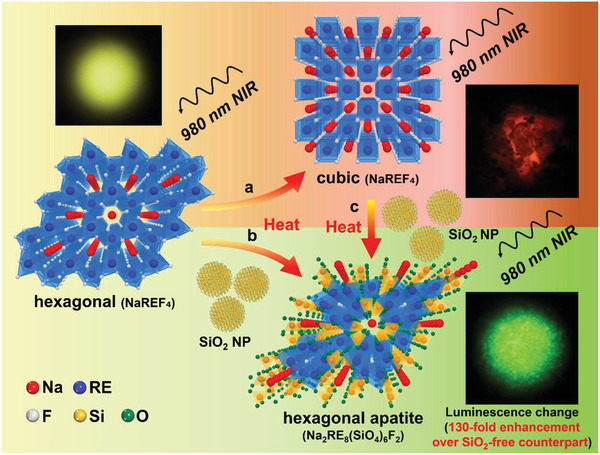
Scheme of the crystal phase transition of UCNs through an annealing process involving SiO_2_. a) Transformation of hexagonal NaREF_4_ to cubic NaREF_4_ with low upconversion efficiency. b) Transformation of hexagonal NaREF_4_ to hexagonal apatite phase by heating it with SiO_2_ NPs with the enhancements of 130‐fold at 550 nm (Er^3+^ energy transfer) with 52‐fold luminance as compared to the cubic phase NaREF_4_. c) Transformation of cubic NaREF_4_ to hexagonal apatite phase with the recovery of the upconversion efficiency. Note that RE indicates rare earth elements (e.g., Y, Gd, Yb, Er, and Tm).

To explore the efficiency of upconversion luminescence process, we prepared yellow (*β*‐NaYF_4_:Gd^3+^, Yb^3+^, Er^3+^ (30/30/2 mol%)) and blue (*β*‐NaYF_4_:Gd^3+^, Yb^3+^, Tm^3+^ (30/18/0.2 mol%)) emissive UCNs‐embedded polyurethane acrylate (PUA) microparticles using an automated DMD‐based mask‐less flow lithography technique with adjusting the presence or absence of SiO_2_ NPs (Figure S3, Supporting Information) and annealed them at different temperature (see Experimental Section for details, For the convenience of quantitative analysis of the effect of SiO_2_ NPs on the luminescent properties of UCNs, photocurable PUA (Mins‐311) was used to synthesize a desired shape of microstructure with good dispersion and transmittance property and to quantitatively measure the luminescence characteristics after thermal process (Figure S4, Supporting Information). Upon annealing at 300 °C, the luminescence color of the UCNs‐embedded microparticles with/without SiO_2_ NPs changed to orange (**Figure** **2**a,b and TGA in Note S2, Supporting Information). As the annealing temperature exceeded 700 °C, a weak red luminescence was observed for the system without SiO_2_ NPs. However, surprisingly, the UCNs‐embedded PUA microparticles containing SiO_2_ NPs exhibited bright green luminescence when the annealing temperature was increased to 900 °C, and the intensity of the emission band at 520 and 550 nm wavelength increased more than 130 times as compared to that of the annealed SiO_2_‐free counterpart (Figure 2d). Moreover, the ratio of 550 and 660 nm luminescence intensities was increased in the UCNs with SiO_2_ NPs after the annealing process (I_550nm_/I_660nm_: 2.54 (annealed UCNs with SiO_2_ NPs) and 0.02 (annealed UCNs without SiO_2_ NPs), Figure S7, Supporting Information). More importantly, the luminance of annealed UCN with SiO_2_ NPs increased ≈52 times than that of SiO_2_‐free UCN (Figure S8, Supporting Information). We supposed that these luminescence properties of post‐processed UCNs are because of the unique energy transfer mechanism of Er^3+^ depending on the crystal phase.

**Figure 2:**
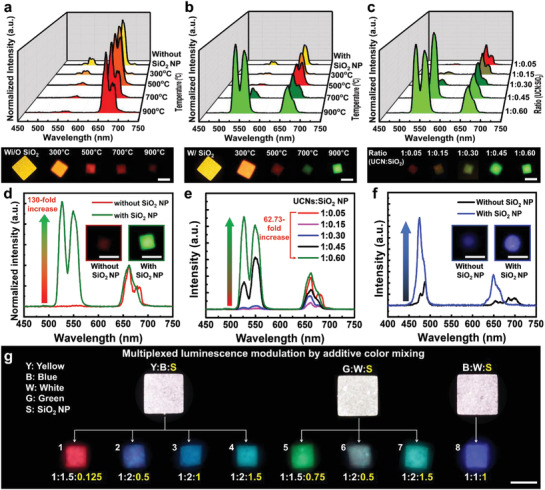
Change in the luminescence color and efficiency of UCNs with/without SiO_2_ NPs after the annealing process. Normalized spectral change (top) and luminescence images (bottom) of UCNs‐embedded microparticles during the annealing process: a) without SiO_2_ NPs, b) with SiO_2_ NPs, and c) upon changing the concentration of SiO_2_ NPs. d) Enhancement of the luminescence intensity of UCNs (*β*‐NaYF_4_:Gd^3+^, Yb^3+^, Er^3+^ (30/30/2 mol%) via SiO_2_ NPs‐involved annealing process at 900 °C. Note that the luminescence spectra with and without SiO_2_ NPs were normalized based on red luminescence intensities (660 nm wavelength). Inset: Luminescence images of UCNs‐embedded microparticles with (green)/without (red) SiO_2_ NPs after the annealing process. e) Luminescence spectral change of UCNs‐embedded microparticles as a function of the concentration of SiO_2_ NPs after annealing at 900 °C. f) Enhancement of the luminescence intensity of UCNs (*β*‐NaYF_4_:Gd^3+^, Yb^3+^, Tm^3+^ (30/18/0.2 mol%) via SiO_2_ NPs‐involved annealing process at 900 °C. Inset: Luminescence images of UCNs‐embedded microparticles with (bright blue)/without (dark blue) SiO_2_ NPs after the annealing process at 900 °C. g) Multiplexed luminescence color modulation by additive color mixing. Note that Y, B, W, and G represent yellow, blue, white, and green UCNs, respectively; S represents SiO_2_ NPs. Scale bar is 100 µm.

We further studied the effect of the SiO_2_ NPs on the spectral changes of UCNs (Composition of the precursor resin is given in Table S2, Supporting Information). When the weight ratio of the SiO_2_ NPs to UCNs was increased from 0.05 to 0.6, the emission band at 550 nm wavelength gradually increased (Figure 2c). The corresponding luminescence intensity at 550 nm increased by ≈60 times as compared to that of system with a SiO_2_/UCNs ratio of 0.05 (Figure 2e). In case of the blue UCNs‐embedded PUA microparticles, luminescence color transition was not observed after annealing but only the intensity of the blue luminescence increased (Figure 2f). We concluded that the weight ratio between Er^3+^‐doped UCNs and SiO_2_ NPs determines the luminescence color transition from red to green, while the Tm^3+^ ions govern the intensity of blue luminescence.

In addition, by combining the aforementioned luminescence property modulation with an additive color mixing method, we achieved multiple luminescence color‐changing strategy for potential utility of multiplexed labeling systems. Four types of UCNs (Y: yellow, B: blue, G: green, and W: white) were blended in different proportions with controlling the SiO_2_ NPs mixing ratio (compositions in Table S3 and luminescence spectra in Figure S9, Supporting Information) and white‐light‐emitting microparticles of YBS, GWS, and BWS were successfully synthesized (Figure 2g). As in Figure 2g, the white luminescence of YBS, GWS, and BWS microparticles were changed to a broad range of multiple colored particles of red, green, and blue with strong luminescence intensity under thermal processing (Luminescence spectra in Figures S10 and S11, RGB values in Table S4, and Note S3, Supporting Information).

To quantify the effect of the annealing temperature on UCNs with/without SiO_2_ NPs, the morphologies of the annealed microparticles containing hexagonal NaREF_4_ UCNs (*β*‐NaYF_4_:Gd^3+^, Yb^3+^, Er^3+^ (30/30/2 mol%), JCPDS file number 01‐072‐4799) with and without SiO_2_ NPs were analyzed at different temperatures. Similar reflections were observed in the X‐ray diffraction (XRD) patterns for both systems (with/without SiO_2_ NPs) annealed at temperatures below 600 °C, indicating that annealing at a low temperature is ineffective for changing the crystal phase of the UCNs (XRD data in Figures S12 and S13, Supporting Information). Upon increasing the annealing temperature to >700 °C (**Figure** **3**a), the crystal phase transitioned to the typical cubic NaREF_4_ phase (JCPDS file number 01‐077‐2042) in the system without SiO_2_ NPs. However, the UCNs in the system containing SiO_2_ NPs transformed to a new crystal phase of hexagonal apatite phase (JCPDS file number 00‐035‐0404) after annealing at 900 °C (Figure 3b). Energy‐dispersive X‐ray spectroscopy (EDS) indicated the Na:F ratio to be ≈1:1, which is consistent with the X‐ray photoelectron spectroscopy (XPS) results (see EDS and XPS data in Figure S14, Supporting Information). Therefore, the stoichiometric composition of the UCNs in the system with SiO_2_ NPs that underwent phase transition was assumed to be Na_2_Y_8_(SiO_4_)_6_F_2_. The difference in the surface morphology depending on the presence or absence of SiO_2_ NPs therefore indicated the evolution of new crystal phases after annealing at 900 °C (Figure 3c–f, and SEM in Figure S15, Supporting Information). Note that PUA has no influence on the morphology, crystal structure, and luminescent property of UCNs with or without SiO_2_ NPs before and after thermal decomposition by the annealing process (Figure S16, Supporting Information). In particular, there exists a large morphological difference at the surface of annealed microparticles with only UCNs and a mixture UCNs and SiO_2_ NPs (Figure 3d,f). The surface of the specimen with SiO_2_ NPs is much smoother than that without them. The smooth morphology of the annealed system with UCNs and SiO_2_ NPs can be ascribed to the enhanced coarsening of grains with diffusional interaction between SiO_2_ NPs and UCNs through Ostwald ripening, whereby Si and O diffuse into the crystal lattice of the UCNs. In general, the diffusional interaction occurs in order to reduce the total energy of the system by reducing the total interfacial area of the particles. Further, the diffusional interaction increases the rate of coarsening through Ostwald ripening, whereby larger particles grow at the expense of smaller ones. According to the Gibbs–Thomson relation, the atomic diffusion occurs from small particles to a larger one, resulting in the growth of the larger particle at the expense of the smaller one (Note S4, Supporting Information). These results are consistent with the results of Fourier transform infrared spectroscopy (FT‐IR) investigations (FT‐IR in Figure S17, Supporting Information). We also confirmed that the cubic UCNs transformed to the hexagonal apatite UCNs as the weight ratio of the SiO_2_ NPs to UCNs (SiO_2_/UCNs) is increased from 0.05 to 0.6 (see XRD data in Figure S18, Supporting Information). Another important aspect of our finding is that the luminescence of cubic UCNs recovered by transforming crystal phase to the hexagonal apatite through annealing process with SiO_2_ NPs (see XRD data in Figure S19 and upconversion luminescence data in Figure S20, Supporting Information). Further, it is clear that the hexagonal apatite UCNs phase is formed regardless of the initial phase of the UCNs (hexagonal NaREF_4_ or cubic NaREF_4_) and two factors, annealing temperature and SiO_2_ NPs, play crucial roles in this process. The SiO_2_ NPs‐involved annealing process described herein is promising for regenerating the lanthanide luminescence at a higher photon energy with higher intensity from cubic UCNs that emit luminescence at lower intensities.

**Figure 3:**
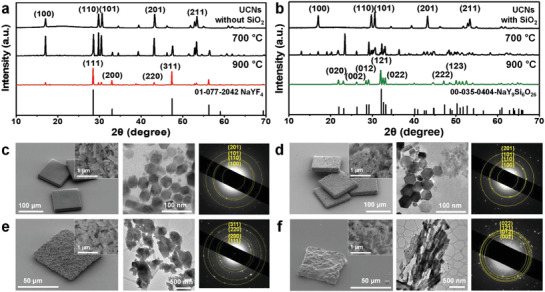
Change in the crystal structure and morphology of UCNs with and without SiO_2_ NPs in PUA microparticles as a function of the annealing temperature. XRD patterns of a) pristine UCNs and b) UCNs with SiO_2_ NPs at different annealing temperatures. SEM images, cross‐sectional TEM images, and SAED patterns of c) pristine UCNs, d) pristine UCNs with SiO_2_ NPs, e) UCNs annealed at 900 °C, and f) UCNs with SiO_2_ NPs annealed at 900 °C.

To understand the luminescence color change of the UCNs corresponding to the crystal phase transition, we considered four factors: crystal field, cross relaxation, nonradiative relaxation, and the electronic properties of the hexagonal NaREF_4_, cubic NaREF_4_, and hexagonal apatite phases of the lanthanide UCNs (**Figure** **4**a). First, the effect of the crystal field on the energy levels of the Er^3+^ activator was investigated (see effective‐operator Hamiltonian calculation in the Experimental Section) and the observed energy level splitting was marginal (10 cm^−1^–10^2^ cm^−1^, Figure S22, Supporting Information). This result indicates that the effect of the crystal field on the color transition is negligible that is consistent with previous reports.^[^ ^40^, ^41^ ^]^ Second, cross relaxation, which could be caused by close distances between the activators (e.g., Er^3+^ or Tm^3+^),^[^ ^25^, ^38^, ^42^, ^43^ ^]^ was verified by comparing the interionic distances between the RE^3+^ ions through the calculated number density of RE^3+^ ions using ab initio molecular dynamics simulation (Figure 4b, density functional theory (DFT) calculation in Experimental Section). We speculate that the cross relaxation of Er^3+^ doped in the host likely occurred more in the cubic NaREF_4_ phase than in the other phases. Third, we found that the cubic phase has higher phonon energy than the hexagonal phase, which implies that nonradiative relaxation is more prevalent in the cubic phase than in the hexagonal phase. Meanwhile, the hexagonal apatite phase showed weaker nonradiative relaxation owing to its low phonon energy compared to that of the hexagonal phase (Figure S25, Supporting Information). Lastly, we studied the electronic properties of the three Er^3+^‐doped crystal phases to trace the energy levels of Er^3+^ between the valence band maximum (VBM) and conduction band minimum (CBM)^[^ ^44^ ^]^ (Figure 4c). In particular, the hexagonal apatite phase showed the lowest energy level of Er^3+^ between the VBM and CBM as compared with those of the other phases, owing to the coordination of the 2*p* state of O^2−^ with Er^3+^, implying that the photons could be more easily excited to the energy state of Er^3+^.

**Figure 4:**
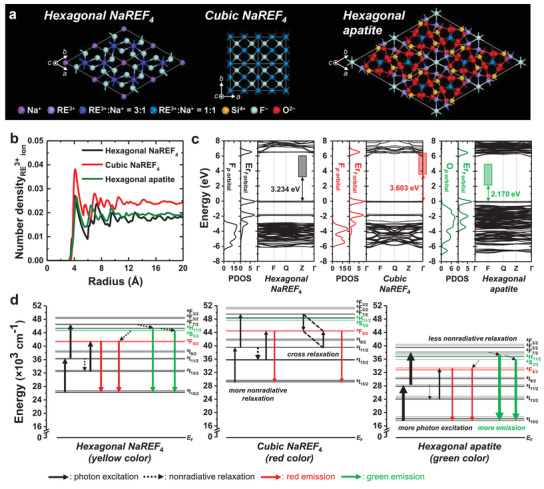
Structural and electronic properties, and the proposed energy transfer mechanism of three crystal phases. a) Schematic of the three types of crystal phases: hexagonal NaREF_4_, cubic NaREF_4_, and hexagonal Na_2_RE_8_(SiO_4_)_6_F_2_ (apatite). b) Number density of rare earth (RE)^3+^ ions within the same radial distance from the reference RE^3+^ ion for the three types of crystal phases. c) Projected density of state (PDOS) and band structure of the three crystal phase systems doped with Er^3+^ ions. Here, 2*p* states were analyzed for F^−^ and O^2−^ ions coordinated to the Er^3+^ ion so as to calculate the lowest energy level of the Er^3+^ ion. The location of the lowest unfilled energy level of Er^3+^ ion was estimated from the energy difference between the maximum of Er^3+^ 4*f* states near the valence band maximum (VBM) and the maximum of F^−^ 2*p* or O^2−^ 2*p* states in the valence band. Each colored box indicates the position of the energy level of Er^3+^ ion in each crystal phase. d) Proposed energy transfer mechanism of Er^3+^ according to the type of phase (hexagonal NaREF_4_, cubic NaREF_4_, or hexagonal apatite). Note that the thickness of the arrows qualitatively indicates the strength of photon excitation, nonradiative relaxation, and emission.

To elucidate the luminescence color changes of the cubic NaREF_4_ and hexagonal apatite phases, energy transfer mechanisms of each UCNs phase are proposed (Figure 4d). One note that the ground ^4^I_15/2_ energy state of Er^3+^ does not have the zero‐energy position because Er^3+^ ion is doped into the crystal structure and the unfilled energy state of 4*f* orbital of Er^3+^ is located between VBM and CBM.^[^ ^44^ ^]^ The location of ground energy state of Er^3+^ (i.e., ^4^I_15/2_) can be estimated from the energy difference between the maximum of Er^3+^ 4*f* states near the VBM and the maximum of F^−^ 2*p* or O^2−^ 2*p* states, which are coordinated to the Er^3+^ ion in each crystal phase, in the valence band. For the cubic phase, cross relaxation (i.e., close interionic distance of Er^3+^) and nonradiative relaxation (i.e., high phonon energy of Er^3+^) induced the red luminescence color. Wang et al. also reported similar cross relaxation induced by the shorter interionic distance of Er^3+^.^[^ ^38^ ^]^ Additionally, the nonradiative relaxation (i.e., ^4^I_11/2_ → ^4^I_13/2_ level) owing to the high phonon energy of Er^3+^ could be another factor for the red luminescence color (Cubic NaREF_4_ in Figure 4d). Evidently, the upconversion luminescence spectrum of the cubic NaREF_4_ indicates the occurrence of cross relaxation through the disappearance of green luminescence between 500 and 600 nm (Figure 2d). Therefore, it is reasonable to consider that cross relaxation and nonradiative relaxation are the major factors determining the yellow‐to‐red luminescence color change. For the hexagonal apatite phase, the green color is primarily induced by the large number of excited photons (i.e., ^4^I_15/2_ level → ^4^F_7/2_ level), followed by weak nonradiative relaxations (i.e., ^4^I_11/2_ → ^4^I_13/2_, ^4^S_3/2_ → ^4^F_9/2_, ^4^F_7/2_ → ^4^H_11/2_, and ^4^H_11/2_ → ^4^S_3/2_) owing to the low phonon energy of Er^3+^ (Hexagonal apatite in Figure 4d). In addition, the upconversion luminescence spectrum also indicates that the ratio between the green and red luminescence intensities for the hexagonal apatite phase increased as compared to that for the hexagonal NaREF_4_ (Figure 2b). Therefore, we conclude that the low energy state and nonradiative relaxations of Er^3+^ in the hexagonal apatite phase are the major factors for yellow‐to‐green luminescence color change. The yellow luminescence of UCNs changed to green in the presence of SiO_2_ NPs, whereas the luminescence color of blue UCNs did not change; instead, the intensity of blue luminescence increased. The blue UCNs (i.e., Tm^3+^‐doped UCNs) with/without SiO_2_ NPs followed the same crystal phase transition (hexagonal to hexagonal apatite or cubic phase) as that of the yellow UCNs (i.e., Er^3+^‐doped UCNs) after annealing at 900 °C (Note S7, Supporting Information).

To provide a proof of concept demonstration of multiple color encrypted particle system, we rationally interweaved the crystal phase transition based luminescence color change and DMD‐based maskless flow lithography technique (details in Note S8, Supporting Information). The utility of our luminescence color transition mechanism was expanded to synthesize binary color‐displaying microparticles (Figure S28, Supporting Information). As shown in **Figure** **5**a, yellow or green luminescent microparticles changed to orange and yellow at 300 °C, respectively. When the temperature reached 900 °C, the color of the area containing SiO_2_ NPs changed to bright green while that of the counterpart changed to red (Figure 5a, bottom).

**Figure 5:**
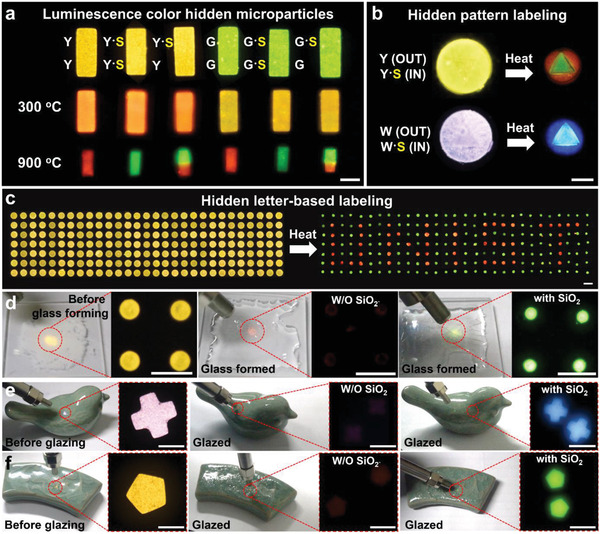
Demonstration of multiple luminescence color changing high temperature labeling systems. a) Luminescence color hidden microparticles. Hidden b) pattern‐ and c) letter‐based labeling system. d) Glass forming and e) and f) ceramic glazing for extreme thermal process monitoring system. Scale bars are 100 µm (a,b,e) and 200 µm (c,d). Note that Y and G represent yellow and green UCNs, respectively; S represents SiO_2_ NPs.

We further applied the aforementioned multiple luminescence color changing strategy to a potential high temperature optical labeling system displaying hidden patterns and letters (for details, see Experimental Section and Figure S29, Supporting Information). As in Figure 5b, by selectively localizing SiO_2_ NPs in the yellow micro‐post, the red triangle pattern was revealed after the thermal process. In addition, by selectively arraying the yellow micro‐posts with/without SiO_2_ NPs using our automated DMD‐based lithography system, a scalable hidden letter encryption system was also realized (Figure 5c).

Lastly, we applied the investigated UCNs luminescence color and intensity changing strategy to conventional thermal processes such as a high‐temperature glass forming and ceramic glazing process (Figure 5d–f). After the glass forming process, the SiO_2_ NPs included yellow luminescence micro‐post array (Figure 5d, left) exhibited bright green luminescence but SiO_2_‐free yellow luminescence micro‐post array emitted almost invisible weak red luminescence after a glass forming process (Figure 5d (middle and right)). White cross and yellow pentagon shaped microparticles without/with SiO_2_ NPs attached to traditional ceramic products such as a pottery bird (Figure 5e) and a pottery spoon rest (Figure 5f). Note that, the white luminescent microparticles with a mixing ratio of Y:B:S (1:2:1) was used, as shown in Figure 2g. After the thermal process, sky blue (hexagonal apatite) cross and bright green (hexagonal‐apatite) pentagon were appeared but SiO_2_‐free counterparts lost their luminescence intensity. Based on the improved luminescent color change with the help of SiO_2_ NPs, we may confirm that the ceramics are the right products that have undergone the proper glazing process at a designed temperature.

Our facile synthetic method can effectively manipulate or tailor the luminescence color of UCNs with high luminescent intensity by involving SiO_2_ NPs to be used in the commercial high temperature thermal processes. We envision that the strategy of SiO_2_ NPs‐promoted luminescence color change of UCNs combined with the described additive color mixing method is a particularly effective means to label information through stepwise alteration of the luminescence color in a high temperature industrial thermal process.

## Experimental Section

##### Synthesis of rod‐shaped β‐NaREF_4_ (RE = Gd, Y, Yb, Er, and Tm) UCNs

Hexagonal phase rod‐shaped NaREF_4_ UCNs were synthesized through a hydrothermal method.^[^ ^13^ ^]^ Briefly, NaOH (0.2 g mL^−1^), ethanol (10 mL), oleic acid (10 mL), a solution of a mixture of lanthanide chlorides (0.2 m RECl_3_•6H_2_O, RE = Gd, Y, Yb, Er, and Tm, 4 mL), and 2 M ammonium fluoride (NH_4_F, 2 mL) were mixed by stirring for 10 min and then transferred to a Teflon‐lined hydrothermal device (lanthanide ion concentrations in Table S1, Supplementary Information). The mixture was then heated at 200 °C for 3 h. Subsequently, the hydrothermal device was cooled naturally to room temperature. The resulting product was collected by centrifugation and washed several times with ethanol and water. The resultant white powder was finally dispersed in cyclohexane.

##### Fabrication of UCNs embedded microparticles with/without SiO_2_

UCNs embedded microparticles with/without SiO_2_ NPs were synthesized by a stop‐flow lithography technique. UCNs with/without SiO_2_ NPs dispersed in a photocurable resin (PUA and photo‐initiator at 9:1 ratio) were flown into a polydimethylsiloxane (PDMS) microfluidic channel and polymerized with patterned UV (365 nm) illumination at 2 mW power through a ×20 objective. Digital micro‐mirror device (DMD)‐based mask‐less lithography was used to control the pattern of UV light. The synthesized microparticles were washed five times with ethanol.

##### Annealing process

PUA microparticles embedded with UCNs and SiO_2_ NPs and covert UCNs microstructures were annealed on sapphire windows (1.5 × 1.5 cm^2^) at a desired temperature (300, 500, 700, or 900 °C) under continuous air flow (mixture of 80% N_2_ and 20% O_2_; 20 sccm) for 1 h in a tube furnace. After the reaction, the samples were cooled to room temperature. To characterize the crystal phase of cubic NaREF_4_ and hexagonal apatite UCNs, the PUA film embedded with UCNs and SiO_2_ NPs was annealed on an alumina boat.

##### Fabrication of microstructures with hidden pattern and letters

Microstructures with hidden pattern and letters were fabricated by controlling the photo‐crosslinking location of SiO_2_ NPs. UCNs with/without SiO_2_ NPs dispersed in a photocurable PUA resin (PUA and photo‐initiator at 9:1 ratio) were coated on an acrylated substrate followed by microparticle fabrication through selective DMD‐patterned UV irradiation using LabVIEW program and an inverted microscope (Nikon Ti‐E). The unpolymerized monomer was rinsed with ethanol. Note that microstructure fabrication was first performed in the presence of SiO_2_ NPs (triangle of Figure 5b, background of Figure 5c) and then in the absence of SiO_2_ NPs (circle of Figure 5b and letters (UNIST) in Figure 5c).

##### Effective‐operator Hamiltonian calculation

To calculate the energy level diagram of the Er^3+^ ion, effective‐operator Hamiltonian calculations were performed. Briefly, the effective‐operator Hamiltonian calculation can be used to construct the energy level diagram of the rare earth (RE)^3+^ ion in the free ion form and under the influence of a crystal field. In this study, the energy level diagram of the Er^3+^ ion was built according to the three crystal phases of the host matrix. The details of the calculation are presented in Note S5, Supporting Information.

##### DFT calculation

DFT‐based ab initio molecular dynamics simulation and geometry optimization were carried out using the CASTEP program.^[^ ^45^, ^46^ ^]^ The details of DFT calculations and model systems are presented in Note S6, Supporting Information.

## Conflict of Interest

The authors declare no conflict of interest.

## Supporting information

Supporting Information
