# Supplementary material for: Multi‐Color Luminescence Transition of Upconversion Nanocrystals via Crystal Phase Control with SiO2 for High Temperature Thermal Labels
Source: Adv Sci (Weinh). 2020 Apr 24;7(11):2000104. doi: 10.1002/advs.202000104 (PMC7284195; doi:10.1002/advs.202000104)
Supplement: Supplementary file 1 — Supporting Information [file ADVS-7-2000104-s001.pdf]

Copyright WILEY-VCH Verlag GmbH & Co. KGaA, 69469 Weinheim, Germany, 2020.

Supporting Information

Multi-Color Luminescence Transition of Upconversion Nanocrystals via Crystal Phase Control with SiO_2 for High Temperature Thermal Labels

Dahye Baek, Tae Kyung Lee, Inkyu Jeon, Se Hun Joo, Subeen Shin, Jaehyun Park, Seok Ju Kang*, Sang Kyu Kwak*, Jiseok Lee*

Materials. Silica nanoparticles (SiO_2 NPs) (13 nm in diameter, US-nano), $GdCl_3$•$6H_2O$ (Aldrich, 99.999%), YCl_3•$6H_2O$ (Aldrich, 99.999%), $YbCl_3$•$6H_2O$ (Aldrich, 99.999%), $TmCl_3$•$6H_2O$ (Aldrich, 99.9%), NH_4F (Aldrich, 99.9%), oleic acid (Aldrich, 90%), PUA (MINS-311 RM, Minuta Tech.), cyclohexane (Aldrich, anhydrous 99.5%), and 2-hydroxy-2-methylpropiophenone (photo-initiator, Aldrich). All chemicals were used as received without further purification.

Characterization. The crystal phase of the products was investigated using a high-power X-ray diffractometer (D/MAX2500V/PC; Rigaku) with Cu $K\alpha$ radiation (λ = 1.5418 Å) at an operation voltage and current of 40 kV and 200 mA, respectively. Thermogravimetric analysis (TGA) was carried out on a Q-500 thermal analyzer at the heating rate of 10 °C min^{-1} over the temperature range of 50–900 °C under an air flow. The morphologies of the samples were examined using a scanning electron microscope (S-4800, Hitachi High-Technologies) after coating the samples with Pt. Bio-transmission electron microscopy was carried out on a JEOL JEM-1400 instrument with a field-emission gun operating at 120 kV. High-resolution transmission electron microscopy was carried out on a JEOL JEM-2100F instrument with a field-emission gun operating at 200 kV to examine the crystallinity of the UCNs and obtain the diffraction pattern. Energy-dispersive X-ray spectroscopic (EDS) mapping of UCNs for the examination of the dopant materials was performed on a HR-TEM instrument. X-ray photoelectron spectroscopy (K-alpha) was used for analyzing the chemical composition of

UCNs. Fourier-transform infrared spectroscopy was carried on a Varian spectrophotometer to determine the vibrational frequencies of Si-O-Si bonds in the spectral range of 4,000–50 cm^{-1} with an attenuated total reflection (ATR) detector. UV-visible-near infrared spectroscopy (solid) was carried out on a Cary-5000 instrument with a photomultiplier tube (PMT) detector in the wavelength range of 300–1,200 nm for reflectance measurement.

Imaging and spectral analysis of UCNs before and after the annealing process. The synthesized UCNs-embedded PUA microparticles were dropped on a slide glass with an imaging solution (10% polyethylene glycol 200 (PEG 200) in ethanol) and then covered with glass. The luminescence of the UCNs-embedded PUA microparticles was measured using a 2 W 980 nm NIR laser with a customized optical setup having a circular irradiation area (Diameter: 550 μm) (MDL-F-980-5W, Dragon laser). A Nikon D-810 camera with a ×20 objective was used for color-imaging, and upconversion luminescence spectra were recorded using a Nikon Ti-E inverted microscope installed with the photoluminescence spectrometer (QEPRO-FL, Ocean Optics). The annealed UCNs-embedded PUA microparticles on a sapphire window were placed on a microscope stage and their upconversion luminescence was measured. A short pass cut-off filter (750 nm cut-off short pass filter, Semrock) was used to block the high-intensity NIR light used for excitation.

Supplementary Note 1. Characterization of *β*-$NaREF_4$ (RE = Gd, Y, Yb, Er, and Tm)

The synthesized *β*-$NaYF_4$:Gd^{3+} UCNs were characterized by scanning electron microscopy (SEM), X-ray diffraction (XRD), high-resolution transmission electron microscopy (HR-TEM), and energy dispersive spectrometry (EDS) (Figure S1). The SEM images showed that the hexagonal rod-shaped UCNs are 300 nm in length and 70 nm in width. The XRD patterns revealed that the crystal phase of the synthesized UCNs matched well with that of conventional hexagonal $NaYF_4$ UCNs (JCPDS 01-072-4799). The HR-TEM images showed the uniform shape and size of the UCNs (300 nm in length and 70 nm in width) and SiO_2 NPs (13 nm in diameter). EDS mapping of the lanthanide ion-doped *β*-$NaYF_4$:Gd^{3+}/Yb^{3+}/Er^{3+} (30/30/2 mol%) indicated a uniform distribution of Y^{3+}, Gd^{3+}, Yb^{3+}, and Er^{3+} ions in the nanocrystal.

Table S1. Luminescence color according to the molar ratios of lanthanide ion dopants in hexagonal UCNs

Luminescence color	Dopant ion (mol%)				
	Y^{3+}	Gd^{3+}	Yb^{3+}	Er^{3+}	Tm^{3+}
Yellow	38	30	30	2	-
Green	50	30	18	2	-
Blue	51.8	30	18	-	0.2
White	38	30	31.7	0.1	0.2

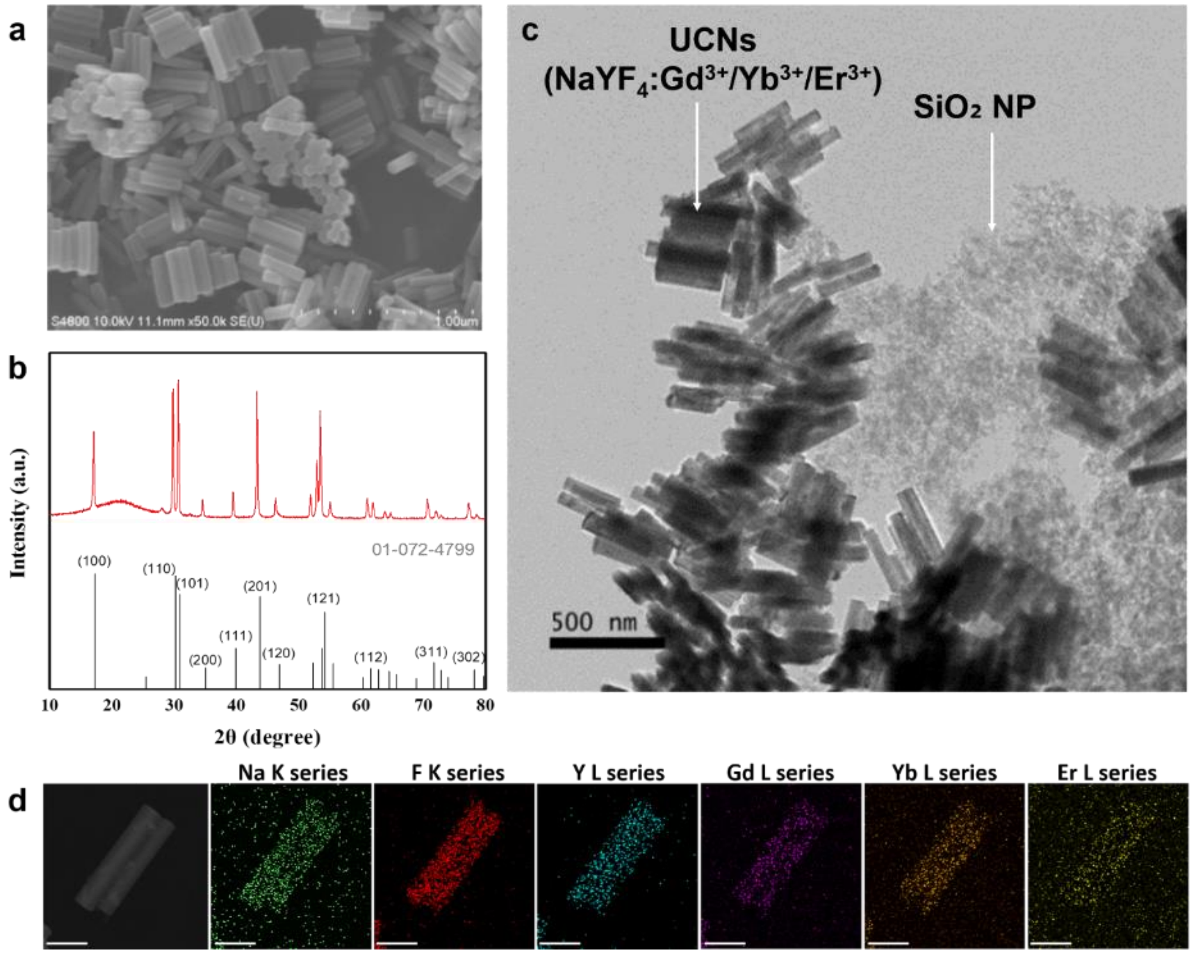

Figure S1. Characterization of hexagonal rod-shaped $NaREF_4$ UCNs. a) SEM image showing the uniform size and shape of nanorods (300 nm in length and 70 nm in width). b) XRD pattern showing hexagonal $NaREF_4$ phase of UCNs matched with reference data (JCPDS 01-072-4799). c) TEM image showing the morphologies of UCNs and SiO_2 NPs, which reveal uniform nanorods (300 nm in length and 70 nm in width) and amorphous SiO_2 NPs (13 nm diameter). d) EDS maps of nanorods obtained from a high-resolution TEM (HR-TEM), which indicate the presence of Y^{3+}, Gd^{3+}, Yb^{3+}, and Er^{3+} ions in a single rod-shaped particle.

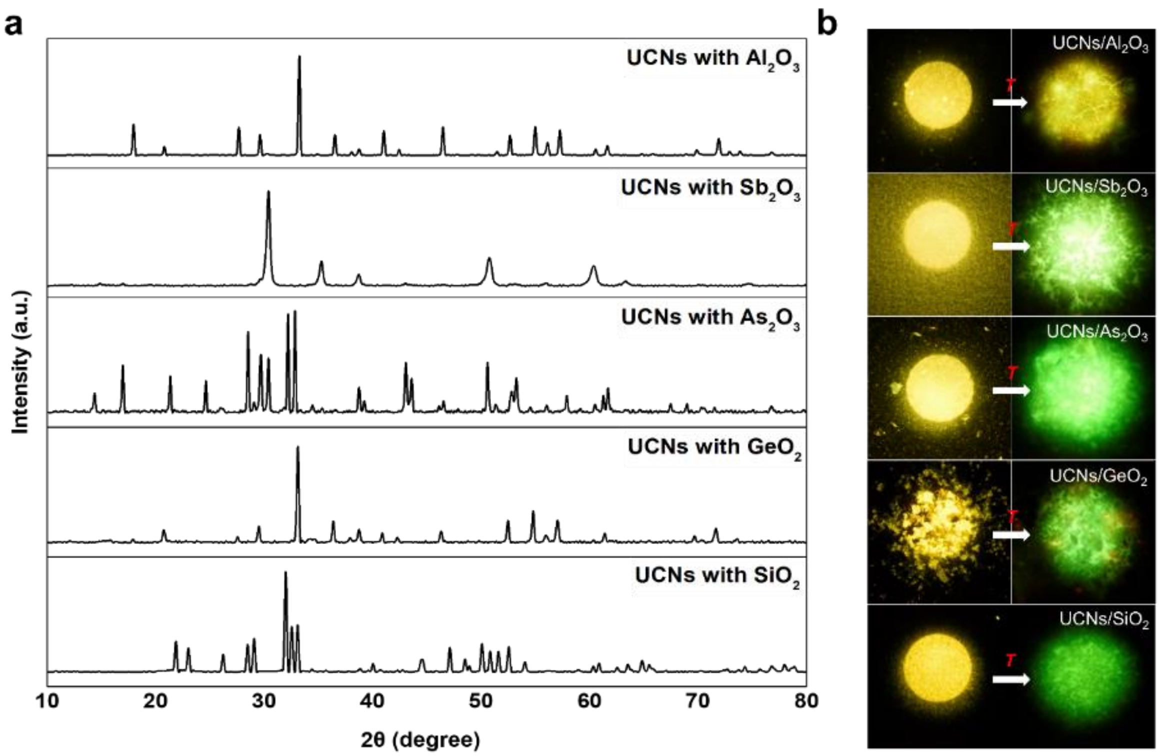

Figure S2. Luminescence image and XRD data of UCNs/metalloid oxide composite compared to those of the UCNs/SiO_2 composite. a) Luminescence images of UCNs/metalloid oxide composites in PUA depending on the type of metalloid oxide: Al_2O_3, Sb_2O_3, As_2O_3, GeO_2, and SiO_2. b) XRD patterns of UCNs/metalloid oxide composites after annealing at 900 °C for 1 h. (The XRD pattern of the above metalloid oxides resulted in several different crystalline phases compared to the hexagonal apatite crystalline phase of UCNs/SiO_2 after the annealing process. These results require further study and are not documented in this manuscript.)

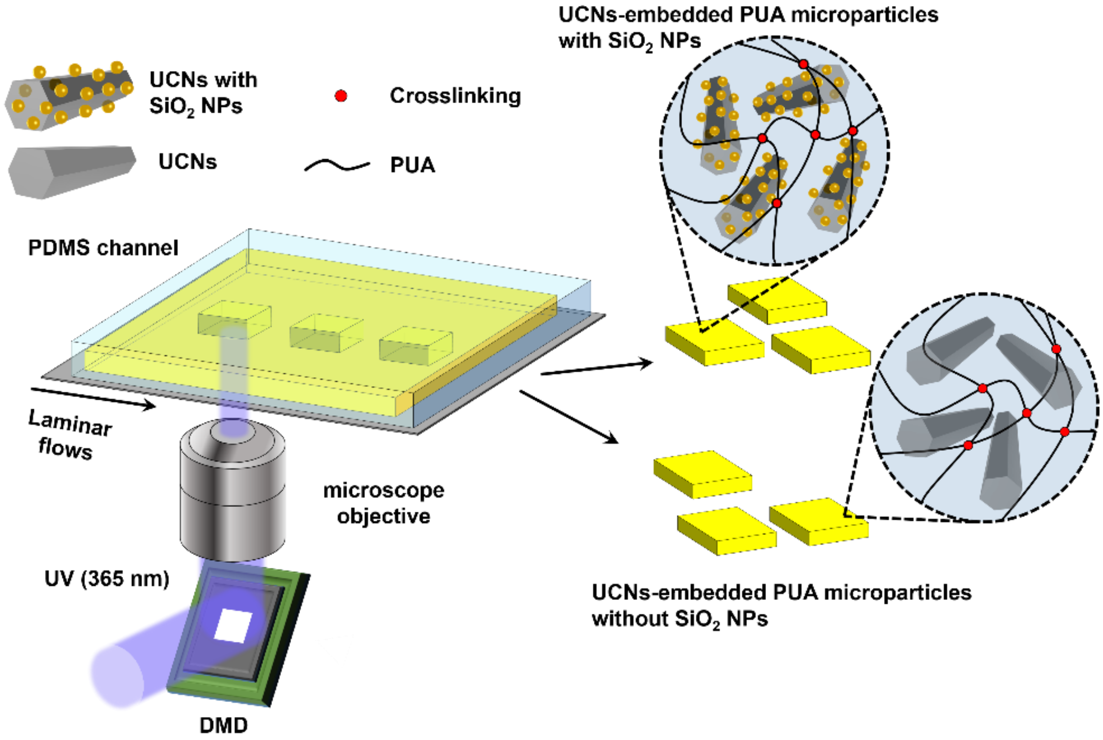

Figure S3. Schematic representation of the fabrication of UCNs-embedded microparticles with/without SiO_2 NPs.

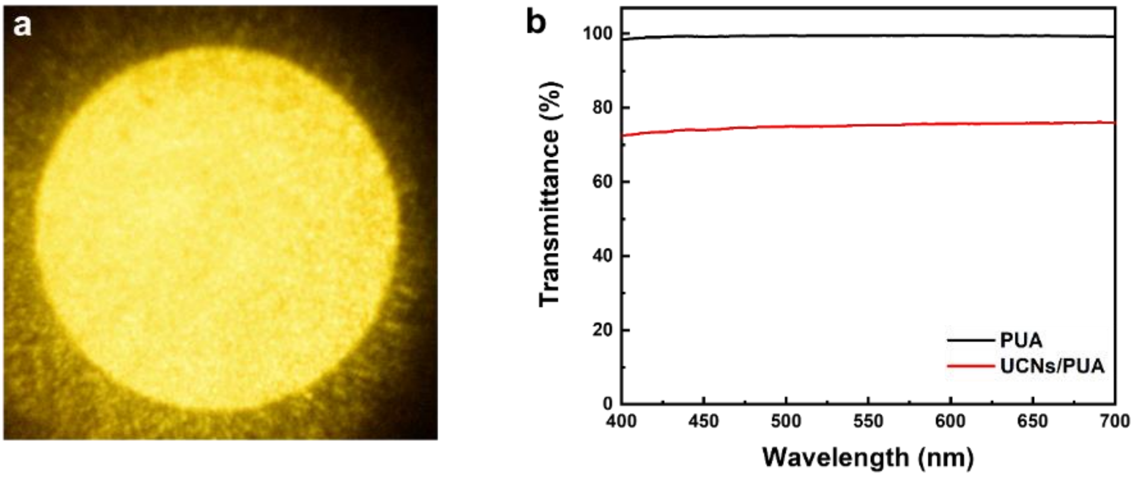

Figure S4. a) Luminescence color image and b) transmittance of UCNs in PUA.

Supplementary Note 2. Analysis of UCNs embedded microparticles with/without SiO_2 by thermal stress

The contraction of PUA microparticles with annealing temperature was confirmed by Thermogravimetric analysis (TGA). Upon annealing at 300 °C, the volume of the microparticles decreased. After annealing at 500 °C, the system with SiO_2 NPs contained 10% more PUA than that of the system without SiO_2 NPs. This is because the added SiO_2 NPs delay the decomposition of carbon. At a higher PUA content, less of the incident NIR light would be absorbed by UCNs, which results in a slight decrease in the luminescence emission intensity (Figure 2a,b, and Figure S5). Upon increasing the annealing temperature to 900 °C, the volume of the microparticles decreased by 80% as compared to that of the original UCNs-embedded microparticles (Figure S6).

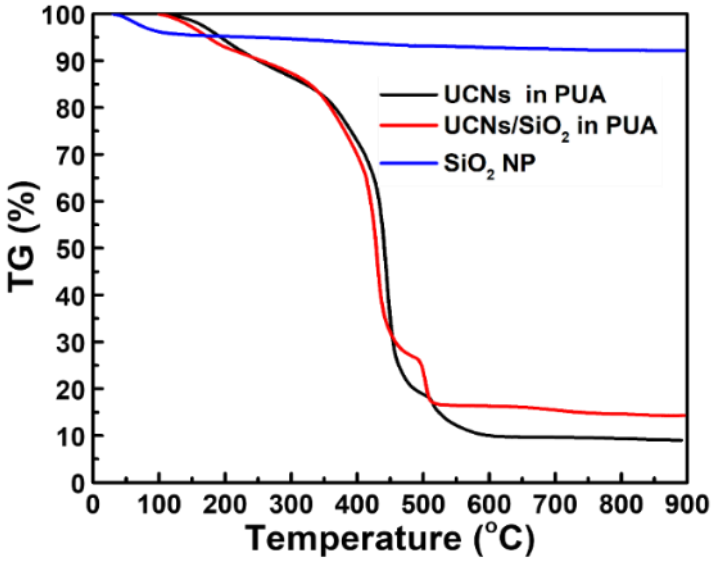

Figure S5. TGA curves of UCNs-embedded PUA film with/without SiO_2 NPs. UCNs-embedded PUA film (60 mg UCNs/600 μL PUA) (black line). UCNs- and SiO_2 NPs-embedded film (60 mg UCNs/30 mg SiO_2/600 μL PUA) (red line). Black and blue curves reveal the decrease in the mass of film at 500 °C. The weight of SiO_2 NPs was maintained after the annealing process (blue line).

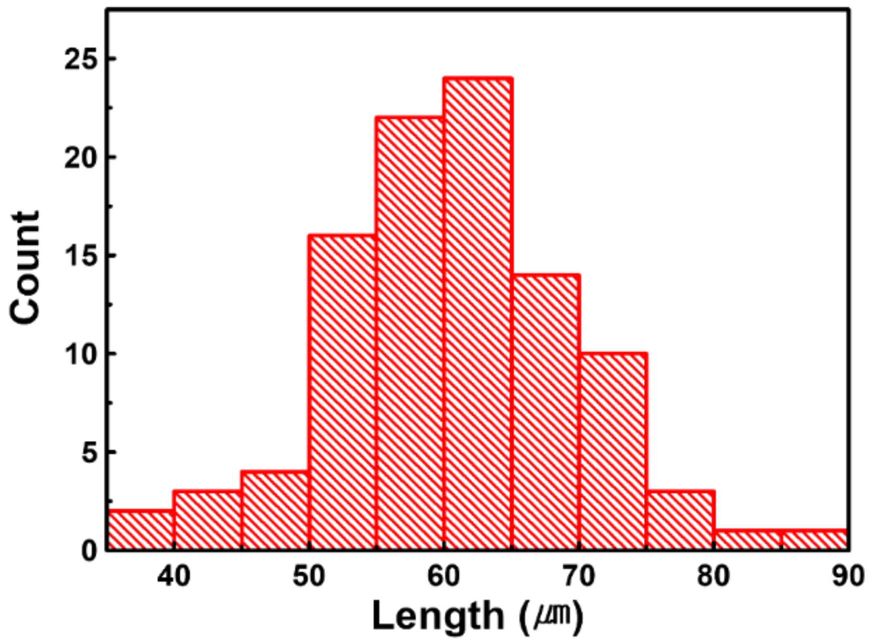

Figure S6. Mean length of UCNs/SiO_2-embedded PUA microparticles after annealing at 900 °C. After annealing, the length of the square-shaped microparticles (*L* = 150 μm) decreased to 65 μm (mean length). The total number of microparticles was 100.

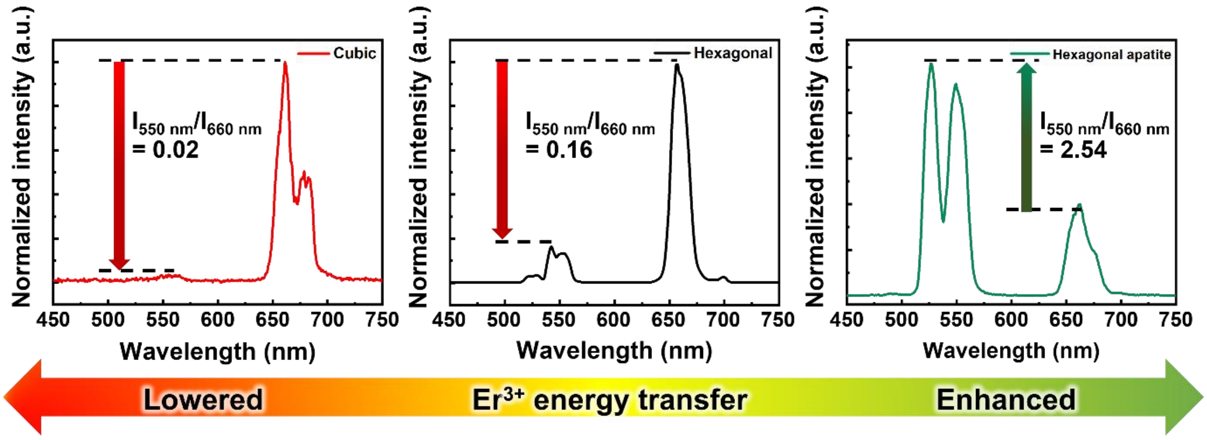

Figure S7. Comparison of the normalized luminescence intensity according to the energy transfer mechanism of Er^{3+}; UCNs without SiO_2 NPs (middle to left), and with SiO_2 NPs (middle to right). RE indicates rare earth elements.

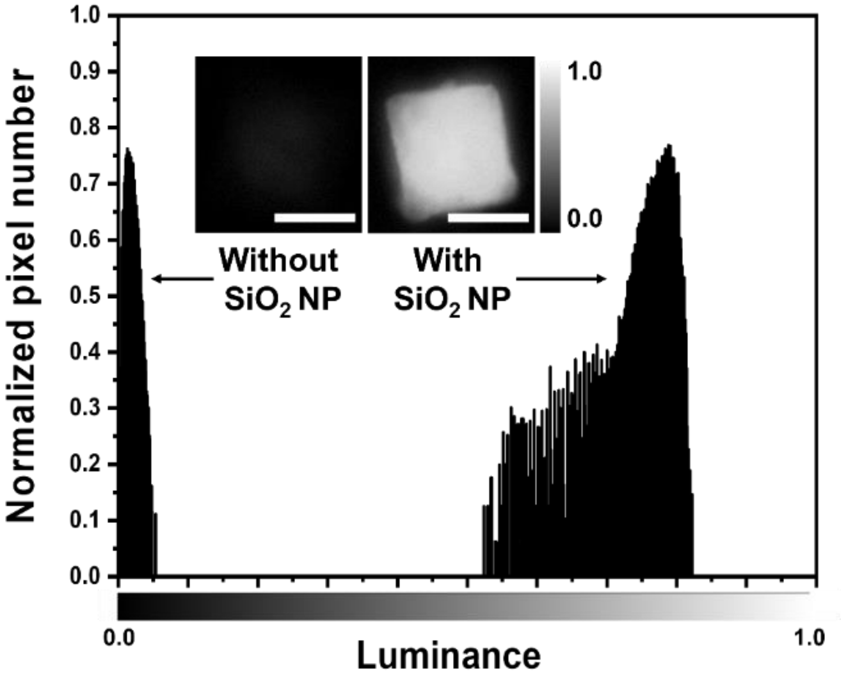

Figure S8. Luminance of UCNs with and without SiO_2 NPs after the annealing process at 900 °C. Inset: standard RGB (sRGB) images of UCNs-embedded microparticles with and without SiO_2 NPs after the annealing process. Scale bar is 50 μm. Note that the luminance is calculated using the standard coefficients for sRGB images as the following equation; Luminance = 0.2126×R+0.7152×G+0.0722×B. The calculated luminance values of UCNs with and without SiO_2 NPs are 0.776 and 0.015, respectively.

Table S2. Concentration of UCNs and SiO_2 NPs in a PUA solution with a photo-initiator

**Mixing ratio ($UCNs:SiO_2$)**	**1:0.05**	**1:0.15**	**1:0.30**	**1:0.45**	**1:0.60**
**SiO_2 (mg)**	3	9	18	27	36
**UCNs (mg)**	60	60	60	60	60
**PUA resin (µl)**	540	540	540	540	540
**Photo-initiator (µl)**	60	60	60	60	60

Table S3. Composition of pre-synthesized UCNs in 100 µL of PUA (PUA and photo-initiator in a 9:1 (v/v) ratio) for multi-color realization

	**1**	**2**	**3**	**4**	**5**	**6**	**7**	**8**
**Yellow (mg)**	4	3.3	3.3	3.3	-	-	-	-
**Blue (mg)**	6	6.6	6.6	6.6	-	-	-	5
**Green (mg)**	-	-	-	-	4	3.3	3.3	-
**White (mg)**	-	-	-	-	6	6.6	6.6	5
**SiO_2 (mg)**	0.5	1.6	3.3	5	3	1.6	5	5

Supplementary Note 3. Multiplexed luminescence color modulation by additive color mixing.

As shown in Figure 2g, when the weight ratio of SiO_2 NPs to Y UCNs in the YBS microparticles was <0.5, the luminescence color changed to red (1 in Figure 2g). However, when the weight ratio of SiO_2 NPs was >0.5, the luminescence color of the YBS microparticles changed to three different greenish-blue colors (2 to 4 in Figure 2g) due to the dominance of the blue transition in the presence of larger amounts of Tm^{3+}. Unlike in the case of the YBS microparticles, the GWS microparticles exhibited bluish-green colors as the weight ratio of SiO_2 NPs to G UCNs in the GWS microparticles was increased from 0.5 to 0.75. We believe that the relative amounts of Er^{3+} ions from both G and W UCNs are larger than those of Tm^{3+} ions, and therefore, green luminescence transition was dominant. Lastly, in the case of BWS microparticles, the luminescence color changed to blue due to the presence of the smallest amount of Er^{3+}; Tm^{3+} is mainly responsible for the color transition

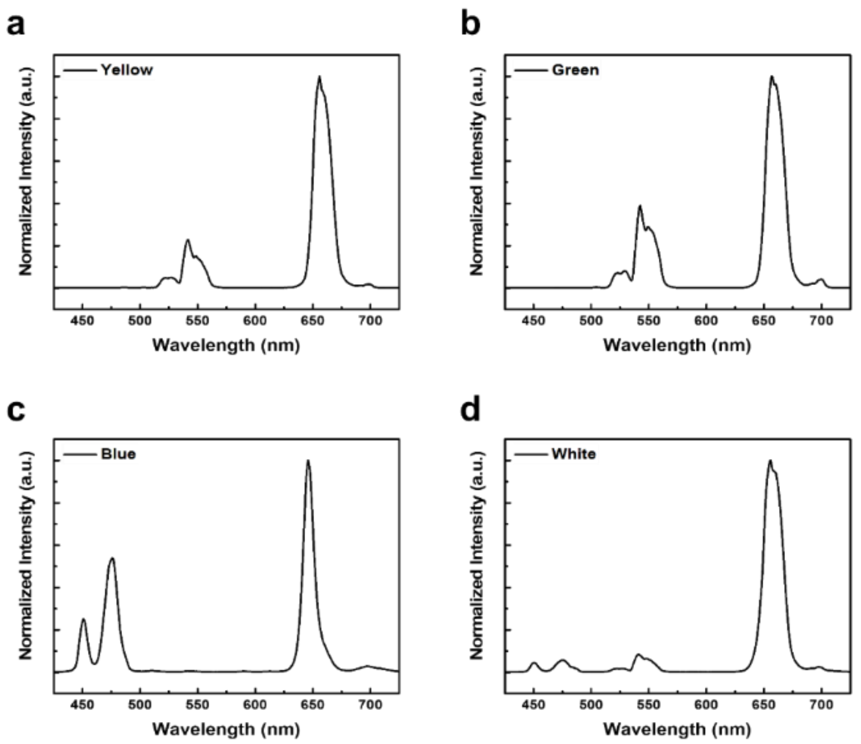

Figure S9. Normalized luminescence spectra of the four types of UCNs with different upconversion luminescence color. Luminescence spectra of a) yellow, b) green, c) blue, and d) white emissive UCNs.

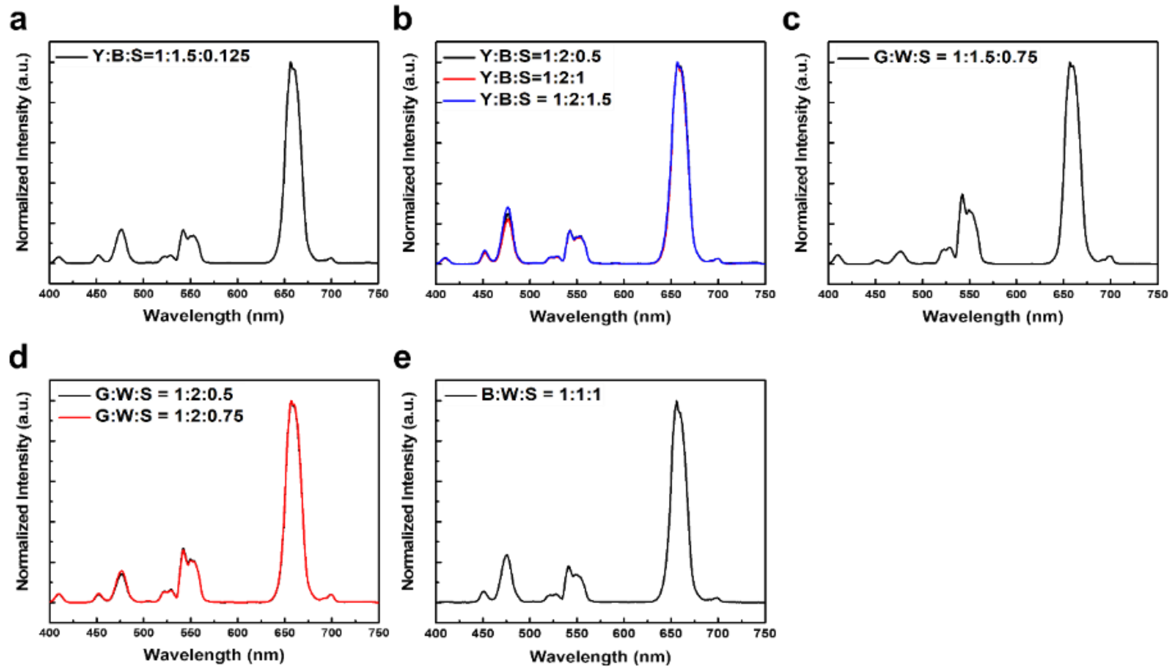

Figure S10. Normalized luminescence spectra of white emissive microparticles. The white emissive microparticles were fabricated by controlling the blending ratio of the different types of UCNs with SiO_2 NPs. Luminescence spectra of a) Y:B:S (1:1.5:0.125); b) Y:B:S (1:2:0.5, 1:2:1, 1:2:1.5); c) G:W:S (1:1.5:0.75); d) G:W:S (1:2:0.5, 1:2:0.75); and e) B:W:S (1:1:1). Note that Y, B, G, W, and S indicate yellow, blue, green, and white UCNs, and SiO_2 NPs, respectively.

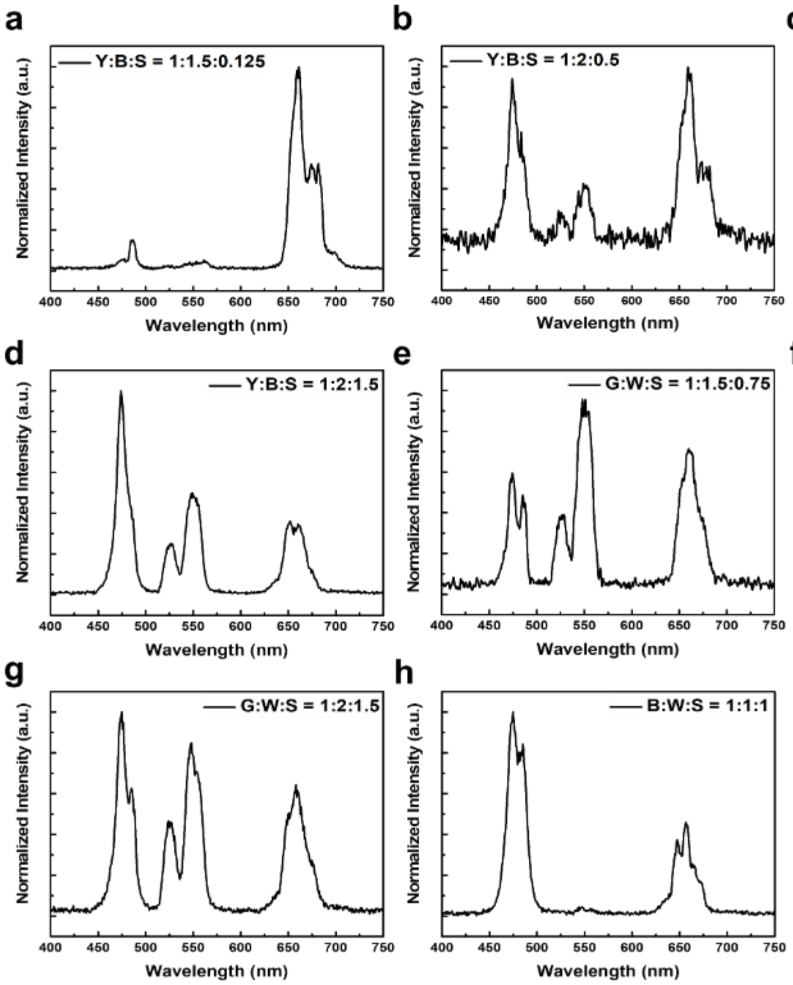

Figure S11. Normalized luminescence spectra of multi-colored UCNs microparticles after annealing. Eight distinctive spectral codes were decrypted from the single white spectral code after the annealing process. Luminescence spectra of a) Y:B:S (1:1.5:0.125); b) Y:B:S (1:2:0.5); c) Y:B:S (1:2:1); d) Y:B:S (1:2:1.5); e) G:W:S (1:1.5:0.75); f) G:W:S (1:2:0.5); g) G:W:S (1:2:1.5); and h) B:W:S (1:1:1). Note that Y, B, G, W, and S indicate yellow, blue, green, and white UCNs, and SiO_2 NPs, respectively.

Table S4. RGB values of the eight distinct luminescence colors of UCNs-embedded microparticles

	1	2	3	4	5	6	7	8
R	211.8	64.3	6.7	9.8	23.1	98.1	44.5	65.6
G	54.4	88.4	102.7	140.6	165.9	131.2	170.3	68.0
B	78.2	153.5	149.2	153.0	96.2	135.3	164.5	245.9

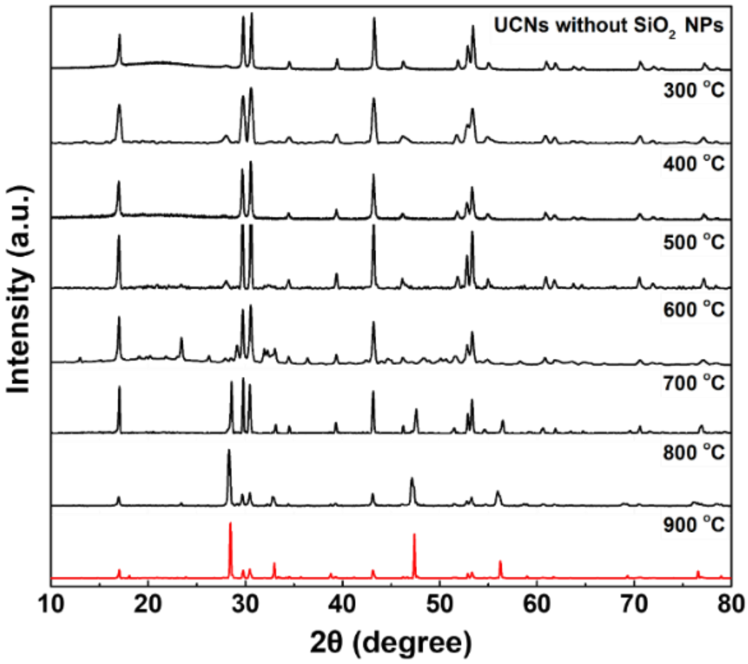

Figure S12. XRD patterns of an UCNs-embedded PUA film heated to different temperatures. The UCNs-embedded PUA film was annealed in an air-purged tube furnace at 300 °C–900 °C for 1 h.

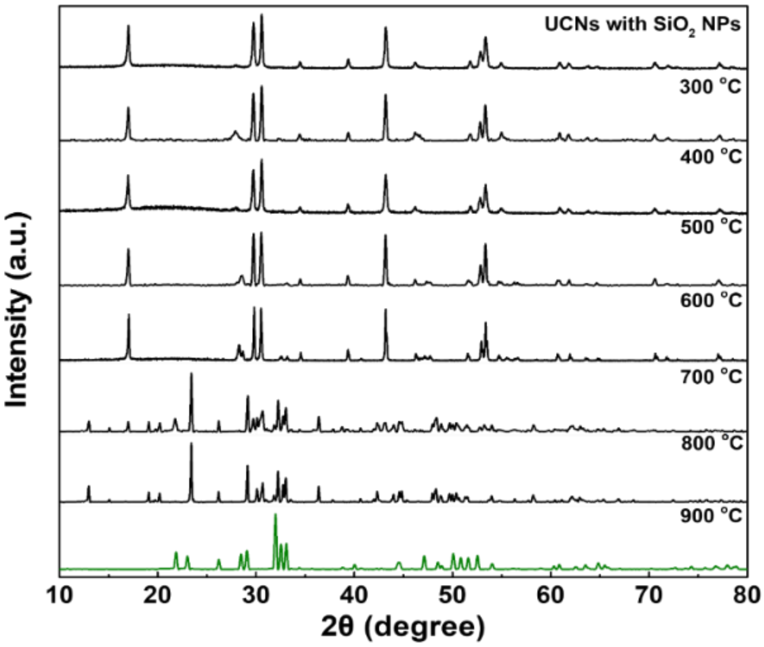

Figure S13. XRD patterns of an UCNs/SiO_2-embedded PUA film heated at different temperatures. The UCNs/SiO_2-embedded PUA film (UCNs:SiO_2 NPs = 1:0.60) was annealed in an air-purged tube furnace at 300 °C–900 °C for 1 h.

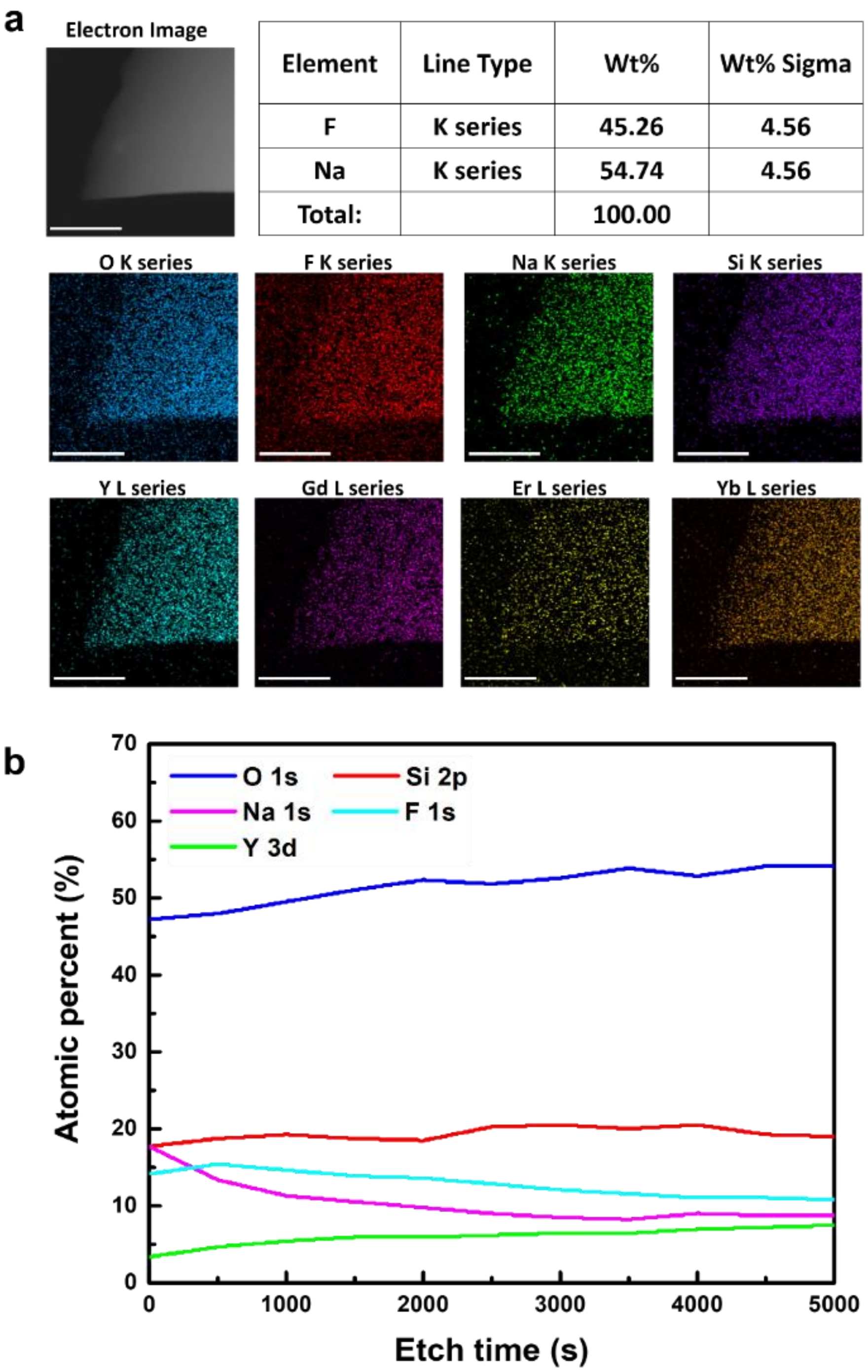

Element	Line Type	Wt%	Wt% Sigma
F	K series	45.26	4.56
Na	K series	54.74	4.56
Total:		100.00	

Figure S14. Results of EDS and XPS analysis of UCNs-embedded PUA film containing SiO_2 NPs after the annealing process. After the annealing process, the ratio between Na and F of UCNs containing SiO_2 NPs was confirmed to be 1:1 by a) EDS mapping and b) XPS analysis. Annealing was conducted at 900 °C for 1 h under continuous air flow (20 sccm). Scale bar: 100 µm.

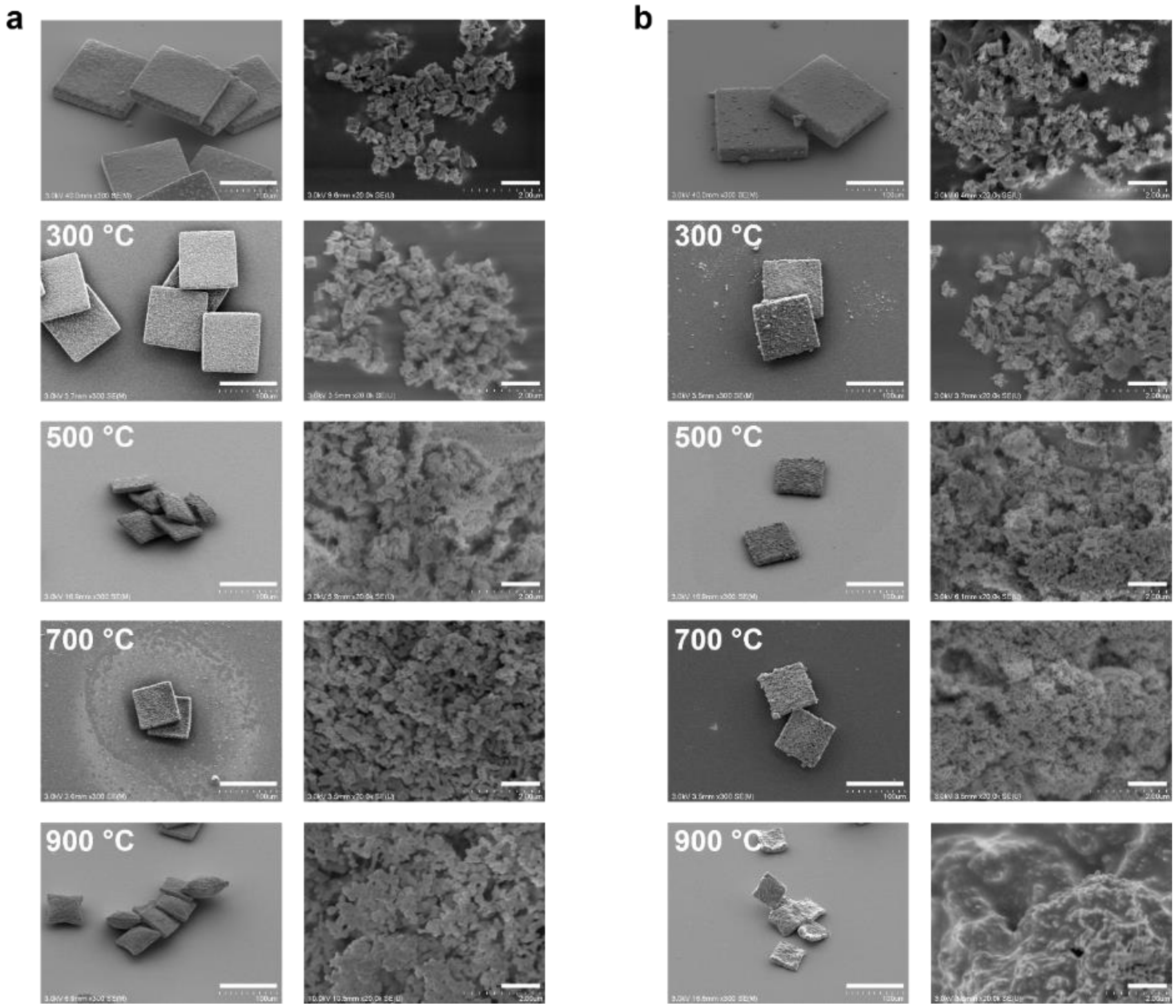

Figure S15. Morphological changes of UCNs-embedded PUA microparticles, according to the annealing temperature. SEM images of a) UCNs-embedded PUA microparticles annealed at 300 °C, 500 °C, 700 °C, and 900 °C for 1 h. b) UCNs- and SiO_2 NPs-(UCNs:SiO_2 = 1:0.60) embedded PUA microparticles annealed at 300 °C, 500 °C, 700 °C, 900 °C for 1 h. From the annealing temperature of 700 °C, a dissolved smooth area appeared on the microparticle surface, and finally, the UCNs and SiO_2 NPs formed an apparently fused surface at 900 °C. (Scale bar: 100 µm (left) and 1 µm (right)).

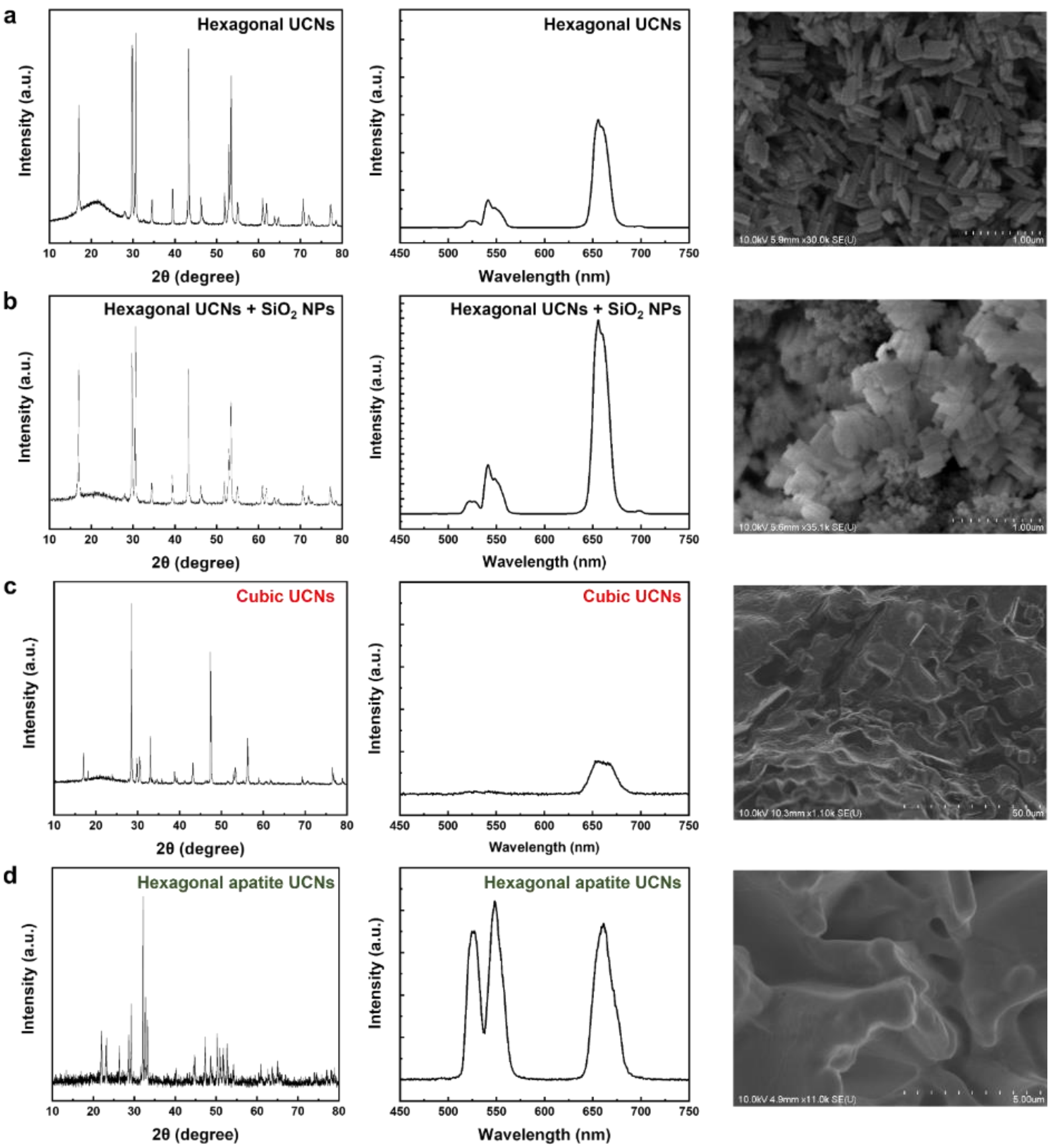

Figure S16. XRD, luminescence spectrum, and SEM analysis of UCNs with and without SiO_2 NPs. Pristine hexagonal phased UCNs a) without and b) with SiO_2 NPs, c) cubic phased UCNs after annealing, and d) hexagonal apatite phased UCNs after annealing. Note all data was obtained without PUA.

Supplementary Note 4. Diffusional interactions between SiO_2 NPs and UCNs during the annealing process

The diffusional interaction increases the coarsening rate through the Ostwald ripening phenomenon, whereby large particles grow at the expense of smaller ones. At the microscopic level, Ostwald ripening occurs due to the atomic migration from small particles to a larger one. Note that the atomic migration can be explained by the Gibbs-Thomson relation,[1–3] which is expressed as follows,

$$C_r = C_\infty e^{\left(\frac{2\gamma V}{kTr}\right)} \tag{1}$$

where C_r is the surface concentration of diffusing atoms of the particle, C_∞ is the surface concentration of atoms in equilibrium with an infinitely large particle, γ is the surface energy of the particle, V is the volume of an adatom, k is the Boltzmann's constant, T is the temperature, and r is the radius of the particle. According to the above equation, small particles have higher C_r than the larger particles. Owing to the concentration gradient, the atomic diffusion occurs from the small particle to a large one, resulting in the growth of the larger particle at the expense of the smaller ones.

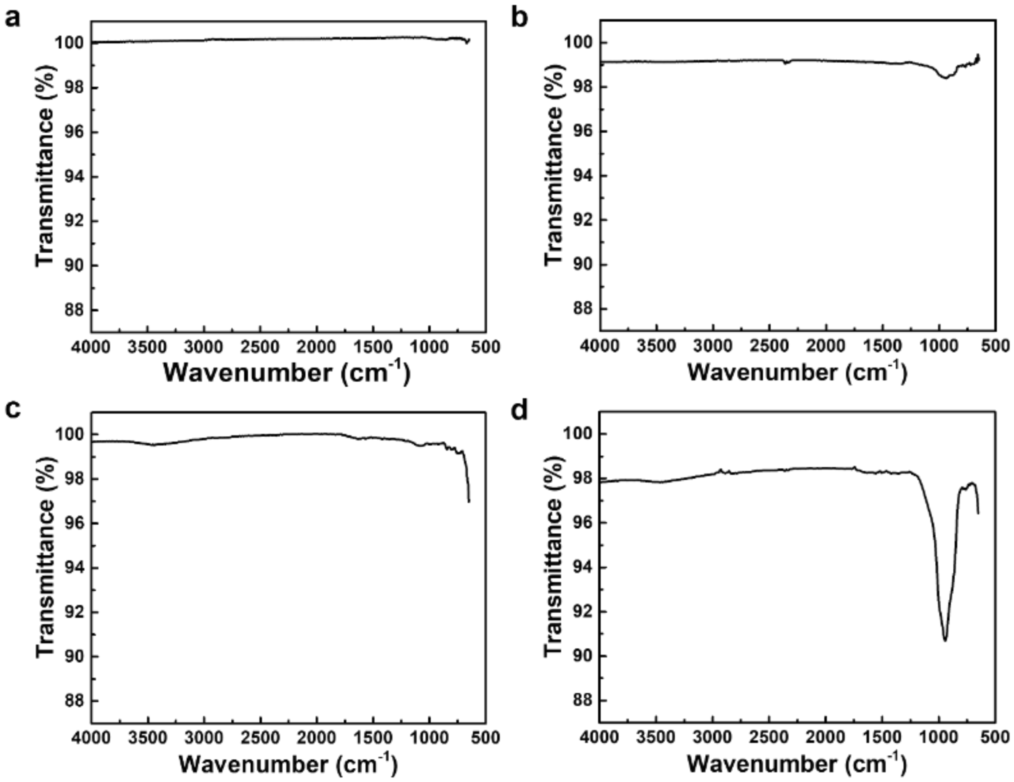

Figure S17. Fourier-transform infrared spectra of an UCNs- and SiO_2 NPs-embedded PUA film. a) UCNs-dispersed PUA film before annealing. b) UCNs-dispersed PUA film after annealing at 900 °C. c) UCNs- and SiO_2 NPs-dispersed PUA film before annealing. d) UCNs- and SiO_2 NPs-dispersed PUA film after annealing at 900 °C. Note that the signal of the Si-O-Si bond at 900 cm^{-1} is more apparent after the annealing process.

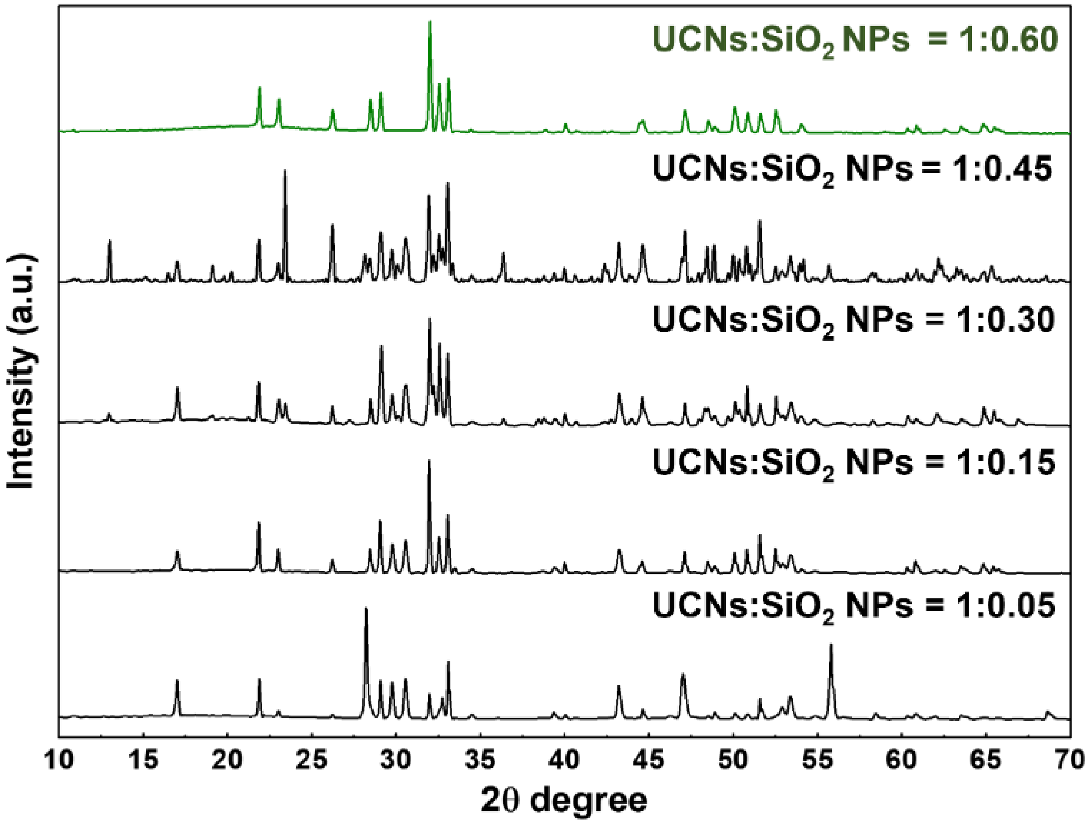

Figure S18. Variation in the XRD pattern according to the concentration of SiO_2 NPs. The UCNs- and SiO_2 NPs-embedded PUA film was annealed in an air-purged tube furnace at 900 °C for 1 h.

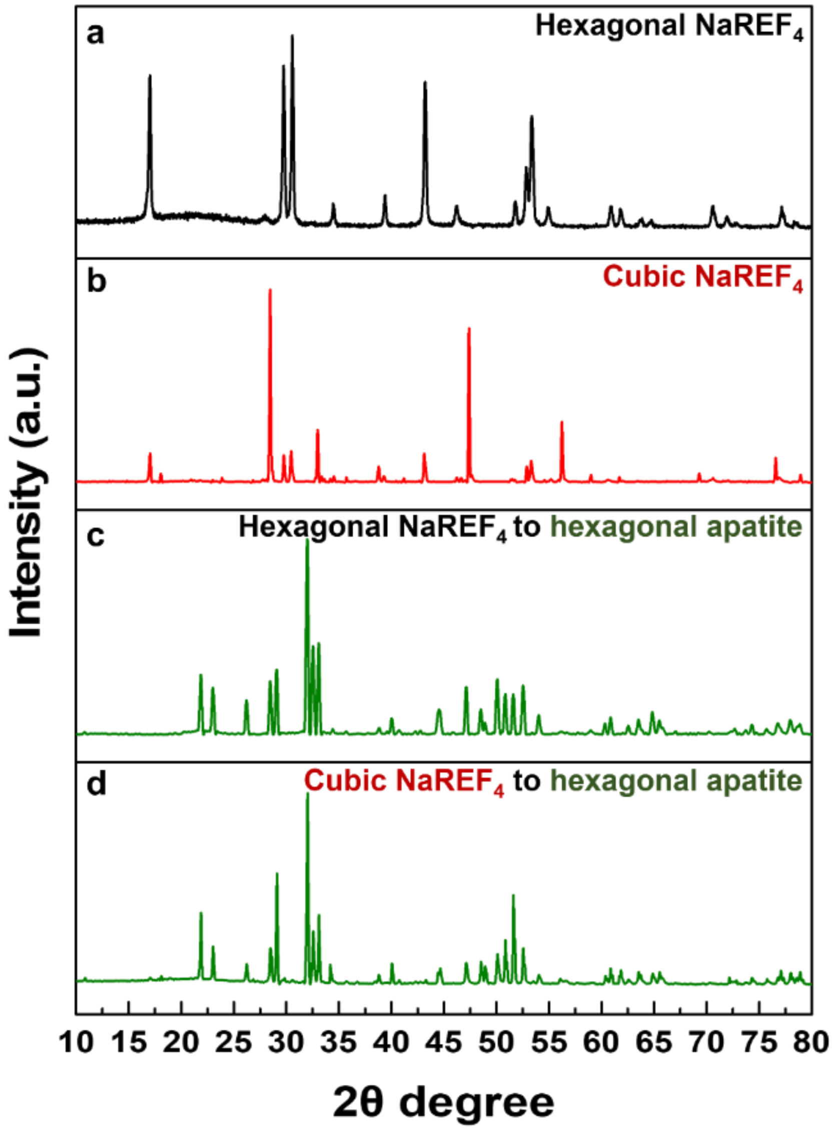

Figure S19. XRD patterns revealing the crystal phase transition of UCNs. XRD pattern of a) pristine hexagonal $NaREF_4$ UCNs (β-$NaYF_4$:Gd^{3+}/Yb^{3+}/Er^{3+}(30/30/2 mol%)) and b) cubic $NaREF_4$ UCNs formed by the annealing of hexagonal $NaREF_4$ UCNs. c) Hexagonal apatite after the SiO_2-involved annealing process. d) Transformation of the annealed cubic $NaREF_4$ UCNs to hexagonal apatite by introducing SiO_2 NPs and annealing. Note that annealing was performed at 900 °C for 1 h under continuous air flow.

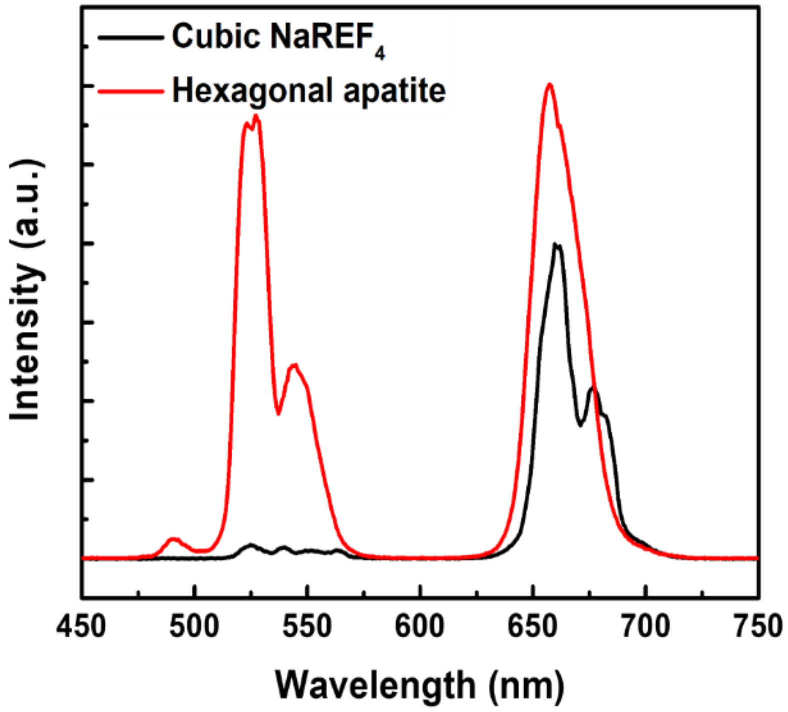

Figure S20. Comparison of the luminescence spectra of cubic $NaREF_4$ and hexagonal apatite UCNs. The black curve is the luminescence spectrum of cubic $NaREF_4$ UCNs obtained by the annealing of hexagonal $NaREF_4$ UCNs (β-$NaYF_4$:Gd^{3+}/Yb^{3+}/Er^{3+}(30/30/2 mol%)) and red curve is the luminescence spectrum of the hexagonal apatite phase UCNs obtained by the annealing of cubic $NaREF_4$ UCNs in the presence of SiO_2 NPs. Note that annealing was performed at 900 °C for 1 h under continuous air flow.

Supplementary Note 5. Construction of the energy level diagram of the Er^{3+} ion

In this study, the energy level diagram of Er^{3+} ion, which was doped in the UCNs with three types of crystal structures, was calculated under the crystal field interaction. To build the energy level diagram of the Er^{3+} ion considering the crystal field effect, the effective-operator Hamiltonian model (Eq. 1) was used.[4]

$$H = H_{FI} + H_{CF} \tag{1}$$

where H_{FI} and H_{CF} represent the effective-operator Hamiltonian models for free ion interactions and crystal field interaction, respectively.

For the free ion interactions, the Hamiltonian model is described as follows:

$$\begin{aligned} H_{FI} = & \sum_{k=0,2,4,6} F^{k} f_{k} + \varsigma_{f} A_{so} + \alpha L(L+1) + \beta G(G_2) + \gamma G(R_7) \\ & + \sum_{i=2,3,4,6,7,8} T^{i} t_{i} + \sum_{h=0,2,4} M^{h} m_{h} + \sum_{k=2,4,6} P^{k} p_{k} \end{aligned} \tag{2}$$

where F^k and ς_f indicate the Coulomb and spin orbit interactions, respectively. f_k and A_{SO} are the angular parts of F^k and ς_f, respectively. α, β, and γ are the parameters associated with the two body correction terms. L is the total orbital angular momentum. $G(G_2)$ and $G(R_7)$ are Casimir's operators for groups G_2 and R_7. T^i and t_i are the three-body interactions and operators, respectively. M^h and P^k are the correlated interactions of magnetic and electrostatic fields, respectively. m_h and p_k are the effective operators of M^h and P^k.

For the crystal field interaction, the Hamiltonian model is as follows:

$$H_{CF} = \sum_{k,q} B_{q}^{k} C_{q}^{(k)} \tag{3}$$

where $B_q^{\ k}$ and $C_q^{(k)}$ are the crystal field parameters and the spherical operators, respectively. For the $C_q^{(k)}$ term, the Wybourne notation is utilized in this calculation.

For the calculation of the Hamiltonian models, the SPECTRA program[5] was used. Based on the peak wavelengths observed in the UV-Vis-NIR absorbance spectra (Figure S21), the parameters of free ion and crystal field interactions were optimized for the three crystal

phases (Table S5). For the crystal field parameters, the site symmetries of Er^{3+} ion, which are D_{3h}, O_h, and C_3 point-group symmetries for hexagonal $NaYF_4:Er^{3+}$, cubic $NaYF_4:Er^{3+}$, and hexagonal $Na_2Y_8(SiO_4)_6F_2:Er^{3+}$ systems, respectively, were considered. Notably, the C_3 point-group symmetry was replaced by C_{3v} symmetry using the descent-of-symmetry method.[4] Based on the optimized parameters, the energy level diagrams of the Er^{3+} ion corresponding to the three crystal phases were constructed (Figure S22).

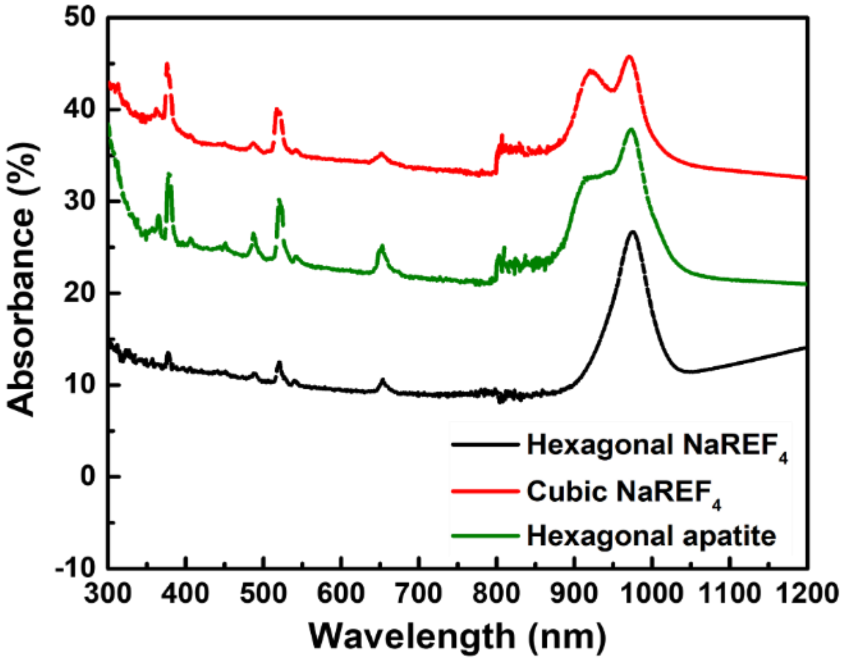

Figure S21. UV-Vis-NIR absorbance spectra according to the crystal phase of UCNs. Absorbance spectra of pristine hexagonal $NaREF_4$ (black line), cubic $NaREF_4$ (red line), and hexagonal apatite (green line) phase UCNs obtained by annealing hexagonal $NaREF_4$ UCNs in the presence of SiO_2 NPs.

Table S5. Optimized parameters of free ion and crystal field interactions for Er^{3+} ions in three types of crystal phases

Parameter	Hexagonal $NaREF_4$ (D_{3h})	Cubic $NaREF_4$ (O_h)	Hexagonal apatite (C_{3v})
F^2	97480	90708	91285
F^4	63728	73292	73758
F^6	56672	51975	52306
ς_f	2247	2307	2268
A	7790	7790	7790
B	−582	−582	−582
Γ	1800	1800	1800
T_2	400	400	400
T_3	43	43	43
T_4	73	73	73
T_6	−271	−271	−271
T_7	308	308	308
T_8	299	299	299
M^0	3.86	3.86	3.86
P^2	594	594	594
B_0^2	−923	-	2246
B_0^4	−839	−1419	4909
B_0^6	67	853	−475
B_3^4	-	-	2719
B_4^4	-	−848	-
B_3^6	-	-	−62
B_4^6	-	1158	-
B_6^6	−24	-	0

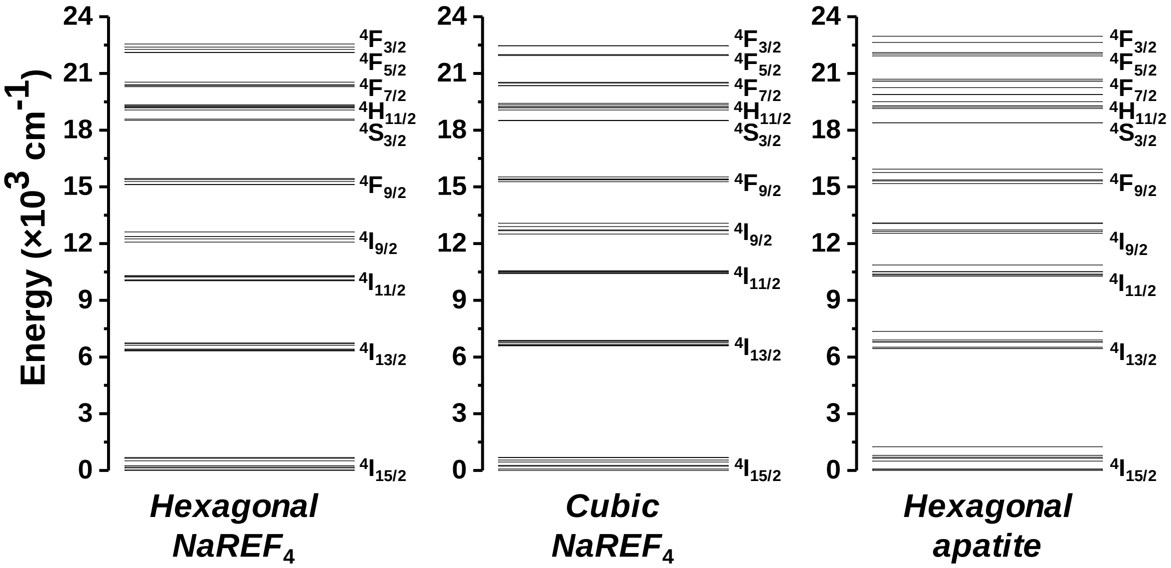

Figure S22. Energy level diagrams of Er^{3+} doped in three crystal phases. Energy level diagrams of Er^{3+} doped in hexagonal $NaYF_4$, cubic $NaYF_4$, and hexagonal $Na_2Y_8(SiO_4)_6F_2$ (apatite), considering the crystal field.

Supplementary Note 6. Computational details for density functional theory (DFT) calculation

A. DFT calculation

Generalized gradient approximation (GGA) with Perdew–Burke–Ernzerhof (PBE) functional,[6] norm-conserving pseudopotential, and a plane-wave basis set were employed in all calculations. The kinetic energy cutoff was set to 780 eV and the Brillouin zone was sampled by 2 × 2 × 2 *k*-point grid with the Monkhorst-Pack scheme.[7] The self-consistent field calculation was performed with electron smearing of 0.5 eV, until the convergence criterion of 2×10^{-6} eV/atom was satisfied. The *ab initio* molecular dynamics (AIMD) simulation was performed in an isothermal-isobaric ensemble (i.e., *NPT* ensemble), where temperature and pressure were controlled by Nose thermostat[8] and Andersen barostat,[9] respectively. Each system was simulated at 298.15 K and 1 atm for 26 ps with a time step of 1 fs. For geometry optimization, the convergence criteria were set to 2×10^{-5} eV/atom for energy change, 0.05 eV/Å for maximum force, 0.1 GPa for maximum stress, and 0.002 Å for maximum displacement, respectively. Moreover, the phonon density of states of Er^{3+} ion was calculated by the finite displacement method (supercell defined by a cutoff radius of 5 Å) and 4 × 4 × 3 (2 × 2 × 3) *k*-point for the Er^{3+}-doped hexagonal or cubic $NaREF_4$ (hexagonal apatite) phase. Lastly, for calculating the electronic structures, Heyd–Scuseria–Ernzerhof (HSE) 06 hybrid functional[10] was used for obtaining accurate band structure and density of states.

B. Model systems

1. Crystal structure of the host nanocrystals

The crystal structures of $NaYF_4$ with $P6_3/m$ and $Fm\bar{3}m$ space groups and $Na_2Y_8(SiO_4)_6F_2$ with $P6_3/m$ space group were used to construct model systems for hexagonal $NaREF_4$, cubic $NaREF_4$, and hexagonal apatite phases of the upconversion nanocrystals, respectively. For the

cubic $NaREF_4$ phase, we employed the unit cell of $NaYF_4$, where two out of four 4*a* Wyckoff positions were occupied by Y^{3+} and the other two 4*a* positions were occupied by Na^+ (Figure S23a). For the hexagonal $NaREF_4$ phase, we used the 1 × 1 × 2 supercell of the $NaYF_4$ unit cell, where two 4*e* Wyckoff positions were occupied by Na^+ and four 2*d* Wyckoff positions were occupied by one Na^+ and three Y^{3+} (Figure S23b). For the hexagonal apatite phase, 4*f* Wyckoff positions were occupied by Y^{3+}, 6*h* Wyckoff positions were occupied by Y^{3+} and Na^+, and 2*a* Wyckoff positions were occupied by F^- (Figure S23c).

2. Crystal structure of UCNs doped with Er^{3+}

The crystal structures of hexagonal $NaREF_4$, cubic $NaREF_4$, and hexagonal apatite systems doped with Er^{3+} were modeled by replacing one of the Y^{3+} atoms by Er^{3+} in the previously constructed model system (Figure S23). All possible doping sites were investigated and the most stable Er^{3+}-doped systems were adopted for the analysis of the phonon density of states and electronic structure (Figure S24).

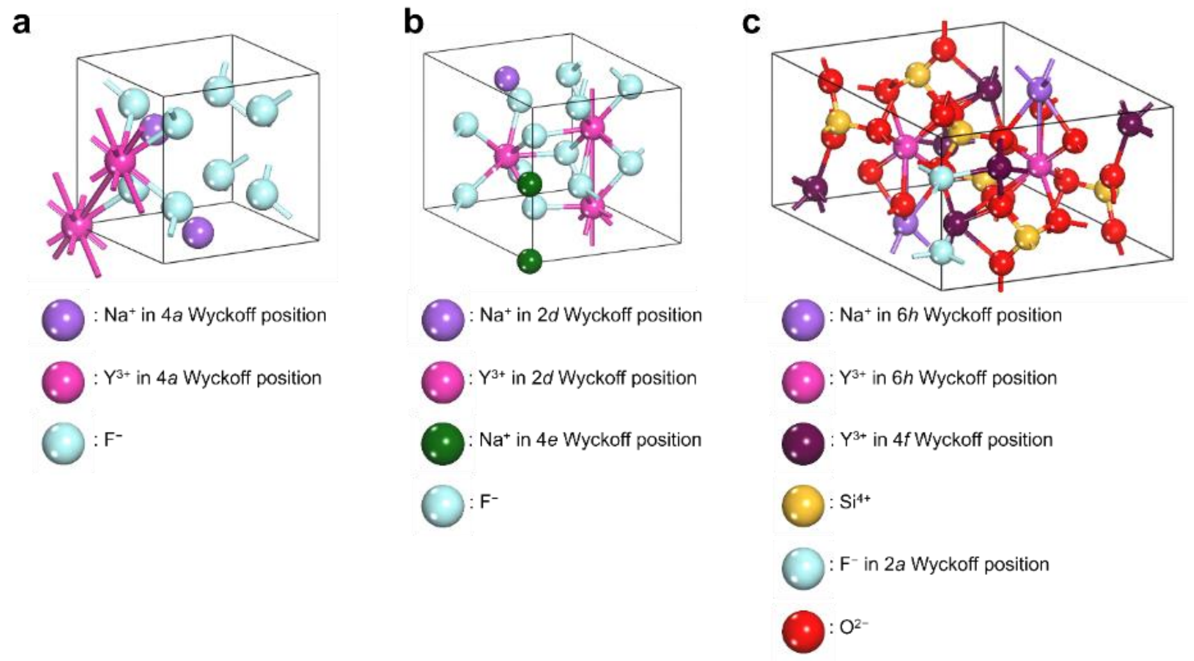

Figure S23. Model systems for the crystal structure of upconversion nanocrystal. Three types of crystal structures, a) cubic $NaREF_4$, b) hexagonal $NaREF_4$, and c) hexagonal apatite phase

structures, are considered. Note that for the hexagonal apatite phase, all possible configurations were investigated where Na^{+} and Y^{3+} ions were placed in 6*h* Wyckoff positions, and the most stable configuration was adopted.

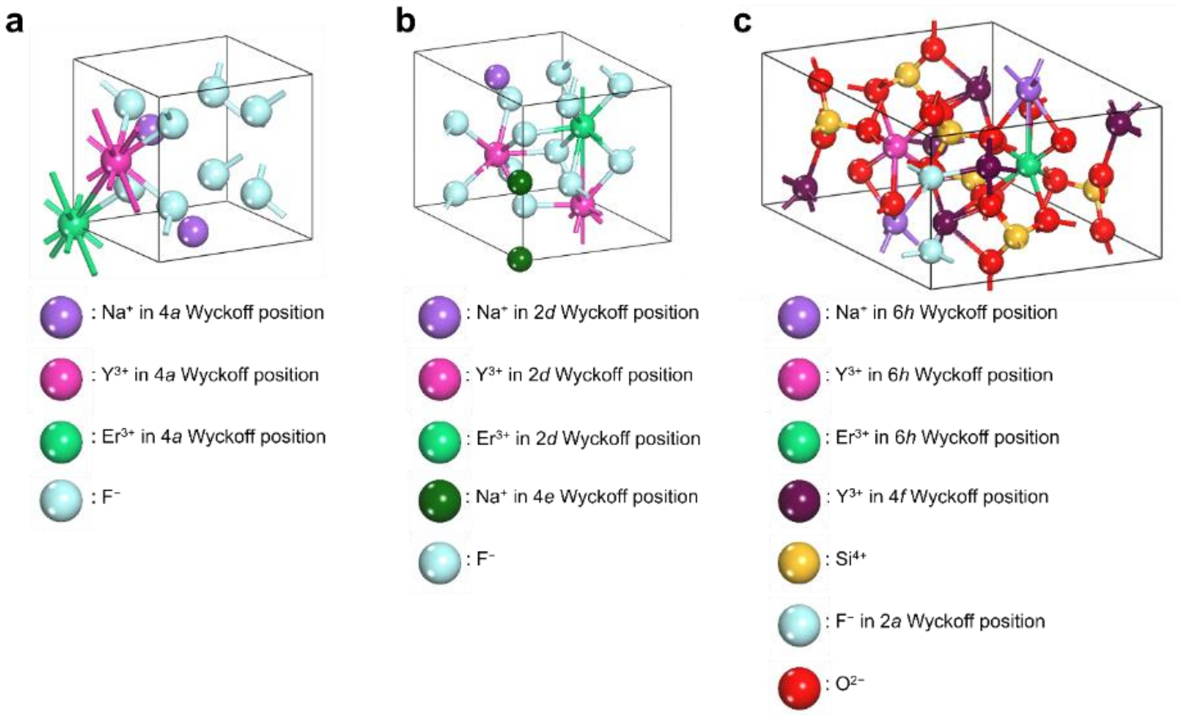

Figure S24. Model systems for the crystal structure of UCNs doped with Er^{3+}. Three types of crystal structures with doped Er^{3+}, a) cubic $NaREF_4$, b) hexagonal $NaREF_4$, and c) hexagonal apatite phase structures, are considered.

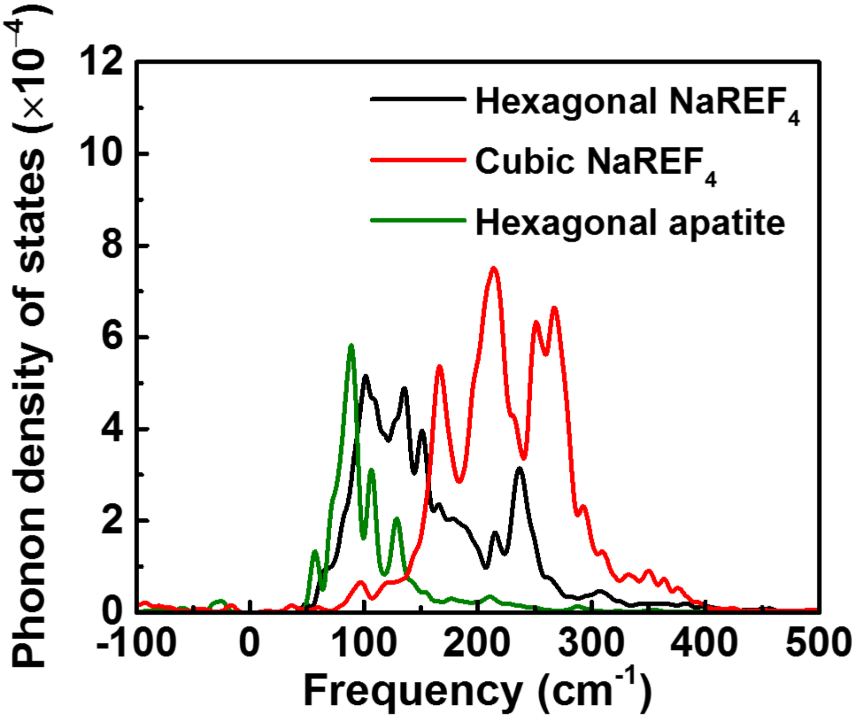

Figure S25. Phonon density of states of Er^{3+} in three types of crystal phases. Phonon density of states of Er^{3+} in Er^{3+}-doped hexagonal $NaREF_4$, cubic $NaREF_4$, and hexagonal apatite

phases. Note that although the imaginary frequency (negative frequency), which indicates structural instability, appeared in the cubic $NaREF_4$ and hexagonal apatite phases, the contribution to the instability of the entire structure was marginal because the intensity was very weak.

Supplementary Note 7. Upconversion luminescence transition of Tm^{3+}-doped UCNs

The blue UCNs (i.e., Tm^{3+}-doped UCNs) showed phase transition from the hexagonal $NaREF_4$ phase UCNs to hexagonal apatite or cubic $NaREF_4$ phase in the presence or absence of SiO_2 NPs, respectively, when annealed at 900 °C (Figure S26). This result indicates that, in our method, regardless of the type of the dopant (e.g., Er^{3+}, Tm^{3+}), the crystal phase of the host matrix of the UCNs plays a crucial role in the tuning of the luminescence of UCNs when subjected to annealing. Based on these results, we could predict that the enhanced luminescence of the system with blue UCNs and SiO_2 NPs after the annealing process is induced by the phase transition of the UCNs (hexagonal $NaREF_4$ phase → hexagonal apatite phase). The difference between blue UCNs and yellow UCNs is the type of emitting ion, which determines the luminescence color. The luminescence color of blue UCNs is determined by Tm^{3+} rather than Er^{3+}. As with the case of the Er^{3+}-doped UCNs, the upconversion luminescence of Tm^{3+}-doped UCNs is influenced by the characteristic of the crystal phase of UCNs, cubic or hexagonal apatite phase (Figure S27). In the cubic phase, processes such as cross relaxation (Figure S27a), less excitation of ions by photons (left side of Figure S27b), and strong nonradiative relaxation (Figure S27c) occur due to the shorter interionic distance of Tm^{3+} ions, higher energy level of Tm^{3+}, and higher phonon energy of Tm^{3+}, respectively, as compared to those in hexagonal apatite phase. The hexagonal apatite phase showed higher photon excitation (Figure S27b, right) and weak nonradiative relaxation (Figure S27c). Based on the energy transfer mechanism of Tm^{3+},[11,12] we speculated that the weak intensity of the upconversion luminescence of the cubic UCNs is induced by the above-mentioned factors: cross relaxation, less excitation of ions by photons, and strong nonradiative relaxation of Tm^{3+} in the cubic phase UCNs. Moreover, for the hexagonal apatite

UCNs, the enhanced luminescence intensities at ~475 nm and ~650 nm in Figure 2f are induced by increased excitation of the ions by photons and weak nonradiative relaxation.

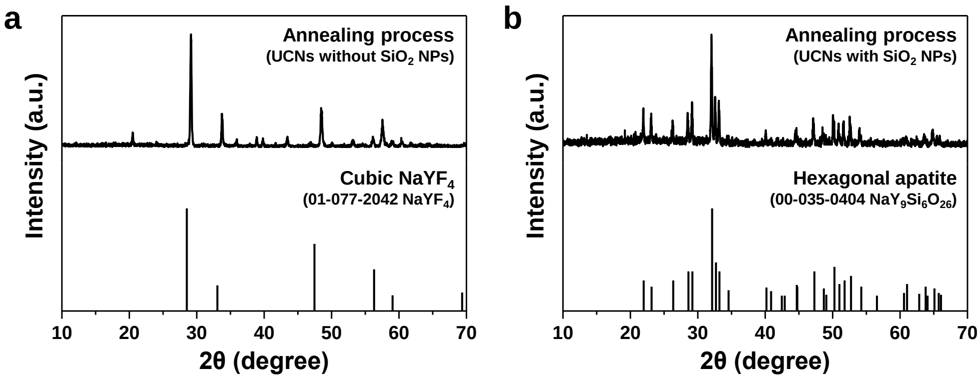

Figure S26. XRD patterns of Tm^{3+}-doped UCNs without/with SiO_2 NPs after annealing at 900 °C: a) Tm^{3+}-doped UCNs (β-$NaYF_4$:Gd^{3+}, Yb^{3+}, Tm^{3+} (30/18/0.2 mol%)) only and b) Tm^{3+}-doped UCNs with SiO_2 NPs.

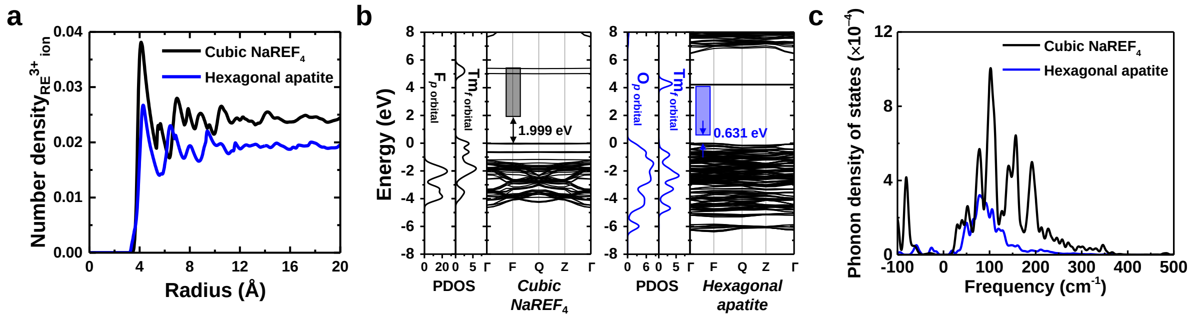

Figure S27. Electronic properties and phonon density of states of Tm^{3+} in cubic $NaREF_4$ and hexagonal apatite phases. a) Number densities of rare earth $(RE)^{3+}$ ions within the same radial distance from the reference RE^{3+} ion for cubic $NaREF_4$ and hexagonal apatite phase UCNs. b) Projected density of states (PDOS) and band structure of the cubic $NaREF_4$ and hexagonal apatite phase UCNs doped with Tm^{3+} ion. Here, 2*p* states were analyzed for F^{-} and O^{2-} ions coordinated to the Tm^{3+} ion to calculate the lowest energy level of the Tm^{3+} ion. The location of the lowest unfilled energy level of Tm^{3+} ion was estimated by the energy difference between the maximum of Tm^{3+} 4*f* states near the valence band maximum (VBM) and the maximum of 2*p* state of F^{-} or O^{2-} in the valence band. Each colored box indicates the position of the energy level of Tm^{3+} ion in each crystal phase. c) Phonon density of states of Tm^{3+} in Tm^{3+}-doped cubic $NaREF_4$ and hexagonal apatite UCNs.

Supplementary Note 8. Multiple luminescence color changing high temperature labeling systems

A. Luminescence color hidden microparticles.

We further expended the utility of our luminescence color transition mechanism to realize binary luminescence color-hidden microparticles (Figure S28). First, as shown in Figure 5a (top), two laminar streams–both streams contained the same colored UCNs (yellow or green) but only one laminar stream contained SiO_2 NPs–were generated in the PDMS channel and photo-crosslinking was carried out in a continuous manner. However, two luminescence colors are distinguished upon increasing the temperature. When the temperature reached 900 °C, the color of the area containing SiO_2 NPs changed to bright green while that of the counterpart changed to red, indicating the generation of binary spectral colored microparticles from singly yellow or green microparticles (Figure 5a (bottom)). Thus, hidden spectral colors could be generated by simply increasing the number of multi-color emitting UCNs in the laminar flow streams and adjusting the presence or absence of SiO_2 NPs.

B. Decryption of hidden pattern and letters.

Microstructures with hidden pattern and letters were fabricated by controlling the photo-crosslinking location of SiO_2 NPs. UCNs with/without SiO_2 NPs dispersed in a photocurable PUA resin (PUA and photo-initiator at 9:1 ratio) were coated on an acrylated substrate, followed by microparticle fabrication through selective DMD-patterned UV irradiation using LabVIEW program and an inverted microscope (Nikon Ti-E). The unpolymerized monomer was rinsed with ethanol. Note that microstructure fabrication was first performed in the presence of SiO_2 NPs (triangle of Figure 5b, background of Figure 5c) and then in the absence of SiO_2 NPs (circle of Figure 5b and letters (UNIST) in Figure 5c). After heat process, the area containing SiO_2 NPs in the yellow micro-post appeared as a green and micro-posts without SiO_2 NPs appeared red under 980 nm NIR laser illumination. Upon following a

similar protocol, the blue micro-post appeared as a triangle with a distinguishable emission intensity difference during the high temperature thermal process (Figure 5b).

C. Glass forming for extreme thermal process monitoring system.

The UCNs embedded yellow luminescence micro-post array with/without SiO_2 NPs was fabricated on the acrylated substrate. For glass forming process, spreading of borosilicate glass powder that is typically used for laboratory glass equipment on the micro-post array, followed by annealing process.

D. Ceramic glazing for extreme thermal process monitoring system.

UCNs-embedded microparticles with/without SiO_2 NPs were fabricated by a stop-flow lithography technique. UCNs with/without SiO_2 NPs dispersed photocurable resin were flown in a microfluidic channel and polymerized by patterned UV irradiation.

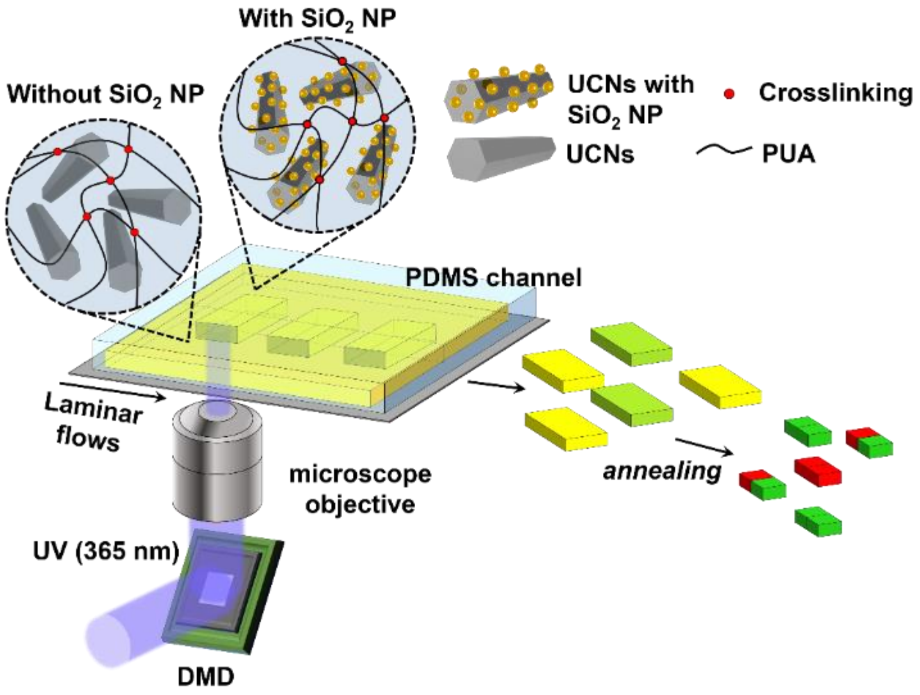

Figure S28. Schematic representation of the fabrication of multi spectral colored microparticles.

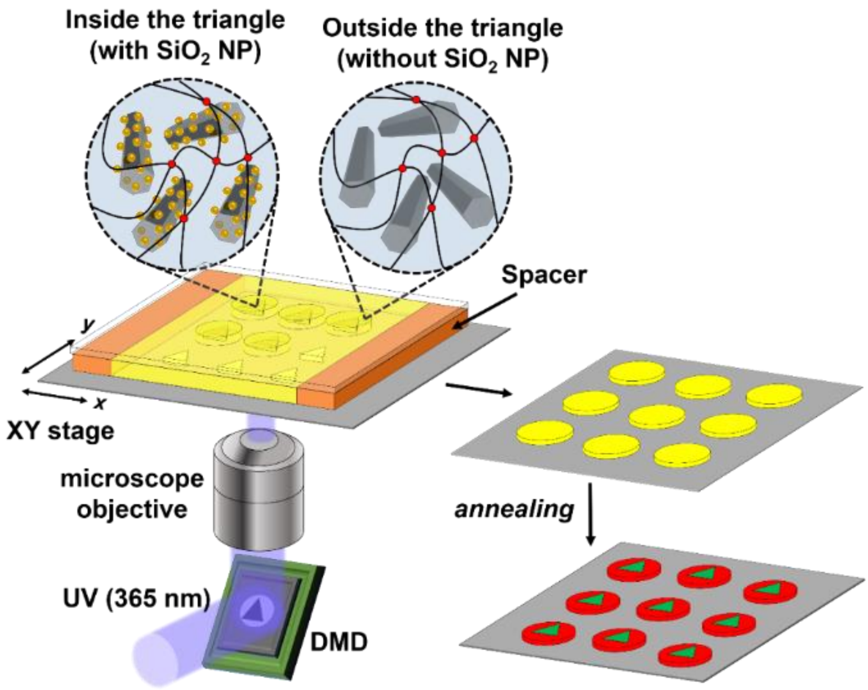

Figure S29. Schematic representation of the fabrication of microstructure array for a hidden pattern labeling system.

Supplementary Note 9. Quantum yield measurement

Quantum yields (QYs) of hexagonal $NaREF_4$ and hexagonal apatite UCNs were measured using an integrating sphere setup at a laser power density of 20 W cm^{-2},[13] as shown in Figure S29.

The quantum yield is calculated as follows:[14]

$$Quantum\ yield\ (QY) = \frac{number\ of\ \ photons\ emitted}{number\ of\ \ photons\ absorbed} = \frac{L_{sample}}{E_{reference} - E_{sample}} \qquad (1)$$

L_{sample} is the emission intensity, $E_{\text{reference}}$ and E_{sample} are the intensities of the excitation laser (980 nm) irradiating the UCNs without/with doped lanthanide ions, respectively.

The QY of an upconverting material is highly dependent on the power density of the NIR laser source because of the nonlinear process of upconversion.[15] Here, QY was measured at a power density of 20 W cm^{-2} of the NIR laser source. As a result, the QY of hexagonal $NaREF_4$ and hexagonal apatite phase UCNs were determined to be ~3.08% and 0.91%, respectively. The QY of hexagonal $NaREF_4$ is consistent with the value reported previously for this material.[14]

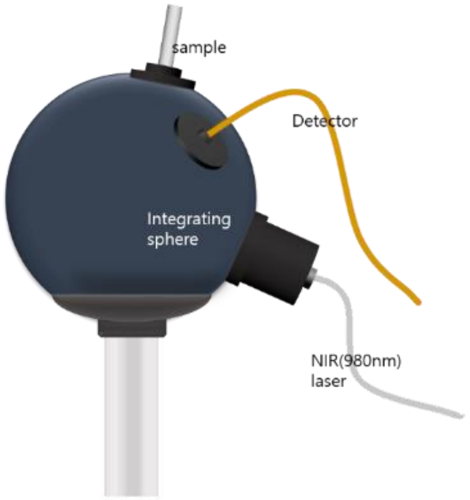

Figure S30. Schematic representation of the integrating sphere setup used for quantum yield measurements.

References

[1] S. B. Simonsen, I. Chorkendorff, S. Dahl, M. Skoglundh, J. Sehested, S. Helveg, *J. Catal.* **2011**, *281*, 147.

[2] P. Wynblatt, N. A. Gjostein, *Prog. Solid State Chem.* **1975**, *9*, 21.

[3] B. K. Chakraverty, *J. Phys. Chem. Solids* **1967**, *28*, 2401.

[4] G. K. Liu, B. Jacquier, *Spectroscopic Properties of Rare Earths in Optical Materials,* Springer Verlag, **2005**.

[5] Spectra Web Site: http://chemistry.anl.gov/downloads/spectra

[6] J. P. Perdew, K. Burke, M. Ernzerhof, *Phys. Rev. Lett.* **1996**, *77*, 3865.

[7] H. J. Monkhorst, J. D. Pack, *Phys. Rev. B* **1976**, *13*, 5188.

[8] S. Nosé, *Mol. Phys.* **1984**, *52*, 255.

[9] H. C. Andersen, *J. Chem. Phys.* **1980**, *72*, 2384.

[10] A. V. Krukau, O. A. Vydrov, A. F. Izmaylov, G. E. Scuseria, *J. Chem. Phys.* **2006**, *125*, 224106.

[11] R. Arppe, I. Hyppänen, N. Perälä, R. Peltomaa, M. Kaiser, C. Würth, S. Christ, U. Resch-Genger, M. Schäferling, T. Soukka, *Nanoscale* **2015**, *7*, 11746.

[12] S. D. Jackson, *Opt. Commun.* **2004**, *230*, 197.

[13] J. -C. Boyer, F. C. J. M. van Veggel, *Nanoscale* **2010**, *2*, 1417.

[14] Q. Liu, Y. Sun, T. Yang, W. Feng, C. Li, F. Li, *J. Am. Chem. Soc.* **2011**, *133*, 17122.

[15] R. H. Page, K. I. Schaffers, P. A. Waide, J. B. Tassano, S. A. Payne, W. F. Krupke, W. K. Bischel, *J. Opt. Soc. Am. B* **1998**, *15*, 996.
